# Theoretical Investigation of the Steric Effects on Alkyne Semihydrogenation Catalyzed by Frustrated Lewis Pairs

**DOI:** 10.1021/acs.jpcc.4c05333

**Published:** 2024-11-07

**Authors:** Allison Zeiss, Jacob Hartman, Sara Kelemen, Jingyun Ye

**Affiliations:** Department of Chemistry and Biochemistry, Duquesne University, Pittsburgh, Pennsylvania 15282, United States

## Abstract

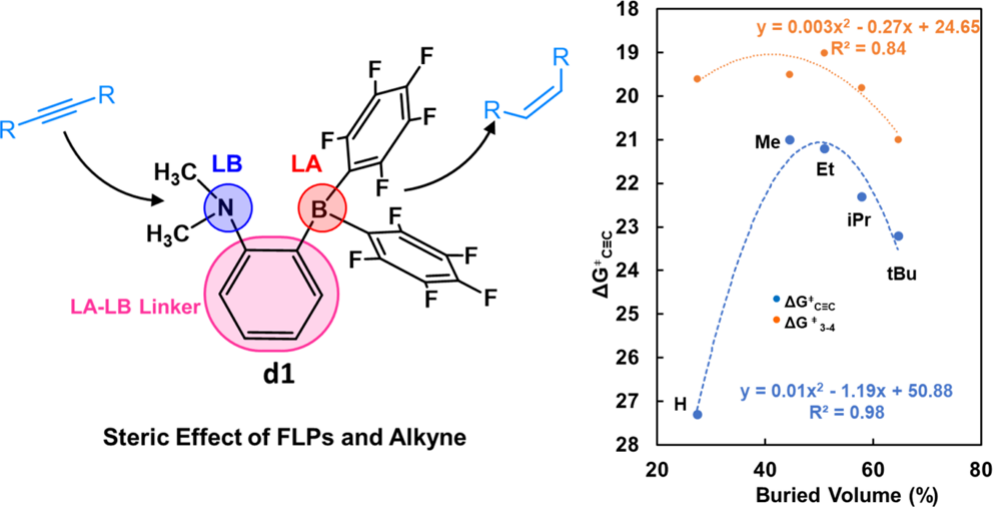

Frustrated Lewis pairs (FLPs) have received increasing attention for offering a distinctive pathway for the activation and conversion of small molecules. Here, we employ density functional calculations to investigate the electronic and steric effects of FLPs and alkynes on the activity toward alkyne semihydrogenation, a crucial reaction in the production of pharmaceuticals and polymers. We investigated the steric effect of FLPs by replacing the LA-LB linker (**d**) of the model catalyst FLP, 1-NMe_2_-2-B(C_6_F_5_)_2_-C_6_H_4_ (**d1**), with various linkers. Additionally, we studied the steric effect of alkynes by varying the substrate from acetylene to but-2-yne, hex-3-yne, 2,5-dimethylhex-3-yne, 2,2,5,5-tetramethylhex-3-yne, and 1,2-diphenylethyne. Our computational results suggest that the overall activity of alkyne and alkene hydrogenation is influenced by both electronic and steric effects when using FLPs with varied LA-LB linkers. To achieve better activity, one could increase the steric hindrance of the LA neighboring environment and reduce the electron density of the LB site of FLPs. Structure–activity relationships for each elementary step involving alkyne semihydrogenation were identified in this work too. In contrast, when the reaction is catalyzed by the same FLP, the activity is solely governed by the steric hindrance of the alkynes. The overall activity exhibits a volcano-shaped trend as a function of the buried volume of the alkynes. FLP (**d1**) shows the highest activity toward hex-3-yne, as predicted by the volcano correlation, while it exhibits lower activity toward alkynes with either less or greater steric hindrance compared to hex-3-yne.

## 1 Introduction

Frustrated Lewis pairs are simply the combination of a bulky Lewis acid (LA) and a bulky Lewis base (LB) sterically precluded from forming classical Lewis acid–base adducts.^1,2^ In this fashion, the unquenched LA and LB sites are available to accept and donate electrons, respectively, offering a distinctive pathway to activate small molecules for catalytic applications.^3−6^ Since the discovery of the first frustrated Lewis pair (FLP) in 2006, numerous FLP systems have been developed, expanding their application beyond reversible H_2_ splitting to the activation and conversion of a variety of small molecules, including CO_2_, N_2_O, NO, SO_2_, imines, alkenes, and alkynes.^7−18^ The electronic and steric effects present in LAs and LBs can both significantly influence the reactivity of FLPs, although the steric effect has received comparatively less attention in studies.^19−28^ The steric effect could arise from either FLPs themselves, the reactant molecules, or a combination of both. The steric factors of FLPs include front strain and back strain engaged in the FLP formation between LA and LB, which could affect the Lewis acid strength, namely, the electronic property of the LA site, ultimately changing the FLP activity.^29^ Steric hindrance in FLPs can induce the dynamic behavior of the LA-LB ring “closed” and “open”, shifting them from “closed” at room temperature to “open” at higher temperatures, thereby enhancing the reactivity of the FLP, as shown in the FLP with B–N heterocycles toward water.^20^ The introduction of weak Lewis basicity by the triptycene scaffold and the steric hindrance of the ortho-substituents enabled the first hydrogenation of unactivated alkenes by FLPs composed of ortho-substituted 9-phosphatriptycene derivatives and tris(pentafluorophenyl)borane B(C_6_F_5_)_3_, overcoming a current limitation in frustrated Lewis pair catalysis.^23^ The steric effect of reactant substrates was also found to impact the reactivity in FLP catalysis. Stephan and co-workers reported an 86% yield in the hydrogenation of Ph_2_C=CH_2_ using [FP(C_6_F_5_)_3_][B(C_6_F_5_)_4_]/pTol_2_NSiEt_3_ as the FLP catalyst at 100 °C and 4 bar of H_2_, while the hydrogenation product yield was only 18% even after 96 h for the more sterically hindered alkene, Ph(2-MeC_6_H_4_)C=CH_2_.^27^

FLPs are a burgeoning field in chemistry, and surprising new reactions catalyzed by FLPs are increasingly being discovered. One such reaction is the chemo- and stereoselective synthesis of alkenes via alkyne semihydrogenation. Alkyne semihydrogenation plays a crucial role in pharmaceutical and polymer production.^30−39^ For example, the selective hydrogenation of acetylene to ethene is vital to the manufacturing industry because trace amounts of acetylene (0.5–3%) in raw ethene are poisonous to the Ziegler–Natta catalysts used for polymerization.^40−43^ To avoid downstream catalyst poisoning, acetylene byproducts in the stream must be reduced to less than 5 ppm.^31,44,45^ Lindlar’s catalyst (Pb-modified Pd/CaCO_3_) has been the choice of catalyst system for industrial semihydrogenation of alkynes to *Z*-alkenes for ∼50 years.^46^ Despite significant progress being achieved, new catalysts predominantly use expensive noble metals (e.g., Pd,^18−40^ Pt,^47^ Ru,^48^ Rh,^49^ Au,^50^ Ag^51^). The best performed single heterogeneous catalyst, Rh_2_Sb/SiO_2_, achieves a 58% yield for diphenylacetylene hydrogenation to E-stilbene, along with the alkane (∼38%) and *Z*-stilbene (∼4%).^52−54^ Therefore, developing low-cost, environmentally friendly, highly active, and selective catalysts for semihydrogenation of alkynes to alkenes is of great interest, but it is still a significant challenge to balance the trade-off between selectivity and activity.

**Figure 1:**
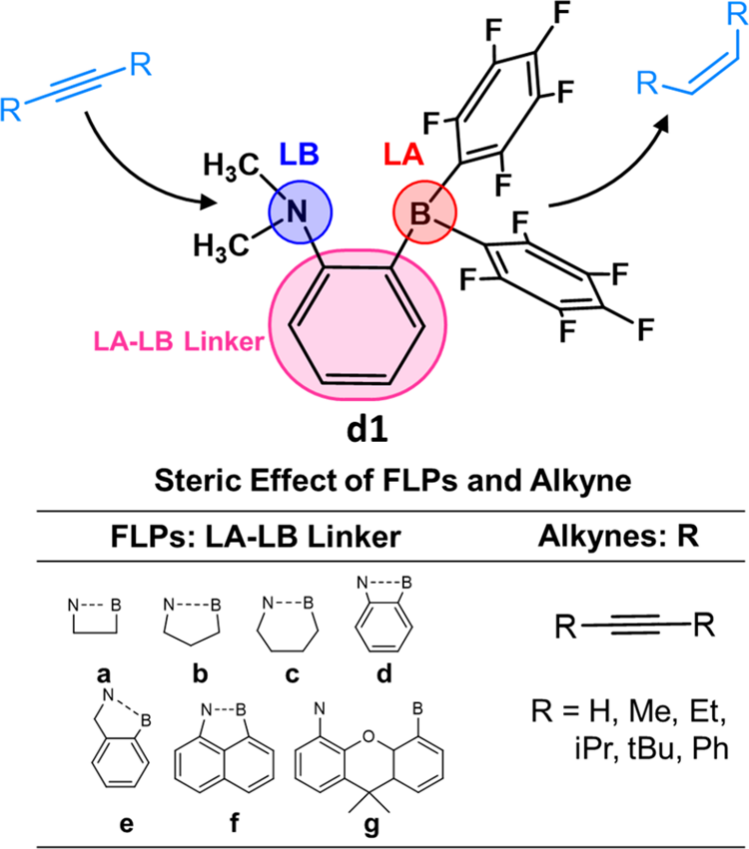
Structure of the 2-[bis(pentafluorophenyl)boryl]-*N*,*N*-dialkylanilines, **d1**, and the steric effect modified by the LA–LB linker (a–g) and *R* group of alkynes.

In 2009, Stephen and co-workers for the first time reported that phenylacetylene reacted with FLPs to give either addition or C–H activation products. Since then, several other FLPs have been shown to activate 1-pentyne,^55^ acetylene,^56^ and terminal alkynes HC≡C–R (R = *n*-Bu, Ph, *t*Bu, SiMe_3_).^57^ In 2013, Repo group and Pápai group reported a FLP based on ambiphilic aminoborane, 1-NMe_2_-2-B(C_6_F_5_)_2_-C_6_H_4_ (**d1**, Figure 1), which shows excellent reactivity for the hydrogenation of internal alkynes (up to 100% conversion).^58^ However, two disadvantages of this FLP catalyst were identified: (1) it is unreactive for alkynes with a terminal triple or double bond, which is attributed to their strong chemical bindings, and leads to the cleavage of the C–H bond of HC≡C–R or H_2_C=CH–R, resulting in catalyst degradation^58^ and (2) the activity decreases with the increase in the steric hindrance of alkynes. For example, the alkyne conversion reduced from 100% when the substrate was 2-butyne to 50% for 1,2-diphenylethyne under the same reaction condition. We have investigated the electronic effect of LA and LB sites on the activity, selectivity, and deactivation of FLPs for acetylene semihydrogenation in our previous work.^59^ However, the steric hindrance of FLPs and the substrates was found to play an important role in this reaction, though it has not been investigated yet.

In the past, research on FLPs has primarily focused on carefully tuning the electronic properties of LA and LB atoms and the steric effects of bulky groups bound to the LA and LB sites. In this work, we aim to understand the steric effects of FLPs arising from the linkers connecting LAs and LBs, as well as the steric effect of alkynes on their activity toward alkyne semihydrogenation using density functional theory (DFT) calculations. We started with the model catalyst FLP, **d1**, and modified its steric hindrance by substituting the LA-LB linker (**d**) with various linkers −C_2_H_4_– (**a**), −C_3_H_6_– (**b**), −C_4_H_8_– (**c**), benzyl (**e**), naphthalenyl (**f**), and dimethyl-xanthenyl (**g**) (Figure 1). This allowed us to adjust the distance between the LA and LB sites and the flexibility of the LA and LB sites. These linkers are selected because they are commonly found in FLPs.^60−65^ On the other hand, the steric effect of alkynes has been investigated by varying the substrate from acetylene (H) to but-2-yne (Me), hex-3-yne (Et), 2,5-dimethylhex-3-yne (iPr), 2,2,5,5-tetramethylhex-3-yne (*t*Bu), and 1,2-diphenylethyne (Ph). The quantitative structure–activity relationships (SARs) for each elementary step involved in alkyne semihydrogenation, as well as for the overall reaction, were identified. These insights will guide the design of FLPs for alkyne hydrogenation and other hydrogenation reactions.

## 2 Computational Methods

Gaussian 16^66^ calculations were performed with the hybrid meta exchange–correlation functional M06-2X^67^ using a def2-TZVP basis set for atoms.^68,69^ The structures of all species were optimized in the gas phase. Harmonic vibrational frequencies were computed to confirm the nature of all intermediates (no imaginary frequencies) and transition-state structures (one imaginary frequency). The gas-phase Gibbs free energies, *G*, were calculated at *T* = 298.15 K and 1 atm pressure by using the harmonic approximation for the optimized structures. The solvation effect of benzene (the solvent used in the experiment^58^) was included by performing single-point energy calculations at the gas-phase geometries using the SMD solvation model.^70^ The relative solution-phase Gibbs free energies were calculated by adding solvation energies to the gas-phase Gibbs free energies. The Cartesian coordinates of all structures and their associated electronic energies, enthalpies, and Gibbs free energies in both the gas phase and in solution are given in the Supporting Information. The energy values reported in the main text are Gibbs free energies (298.15 K, a standard state of 1 atm for gases, and 1 M for solutes) including the solvent effect of benzene. Partial atomic charges were calculated for the gas-phase molecules using the CM5 charge model developed by Truhlar and co-workers.^71^ The buried volumes were calculated using the SEQCROW^72,73^ toolset in ChimeraX.^74^ The occupied volume of a 3.5 Å radius sphere was calculated by defining the center of the sphere to be the center of the LA/LB site of the FLPs or the center of the carbon–carbon triple bond in the alkynes. The UMN radius (the vdW radius from Mantina, Chamberlin, Valero, Cramer, and Truhlar)^75^ of each atom was used and then scaled by a factor of 1.17 to calculate the volume of the sphere occupied by atoms.

## 3 Results and Discussion

### 3.1 Reaction Mechanism and Activity

**Figure 2:**
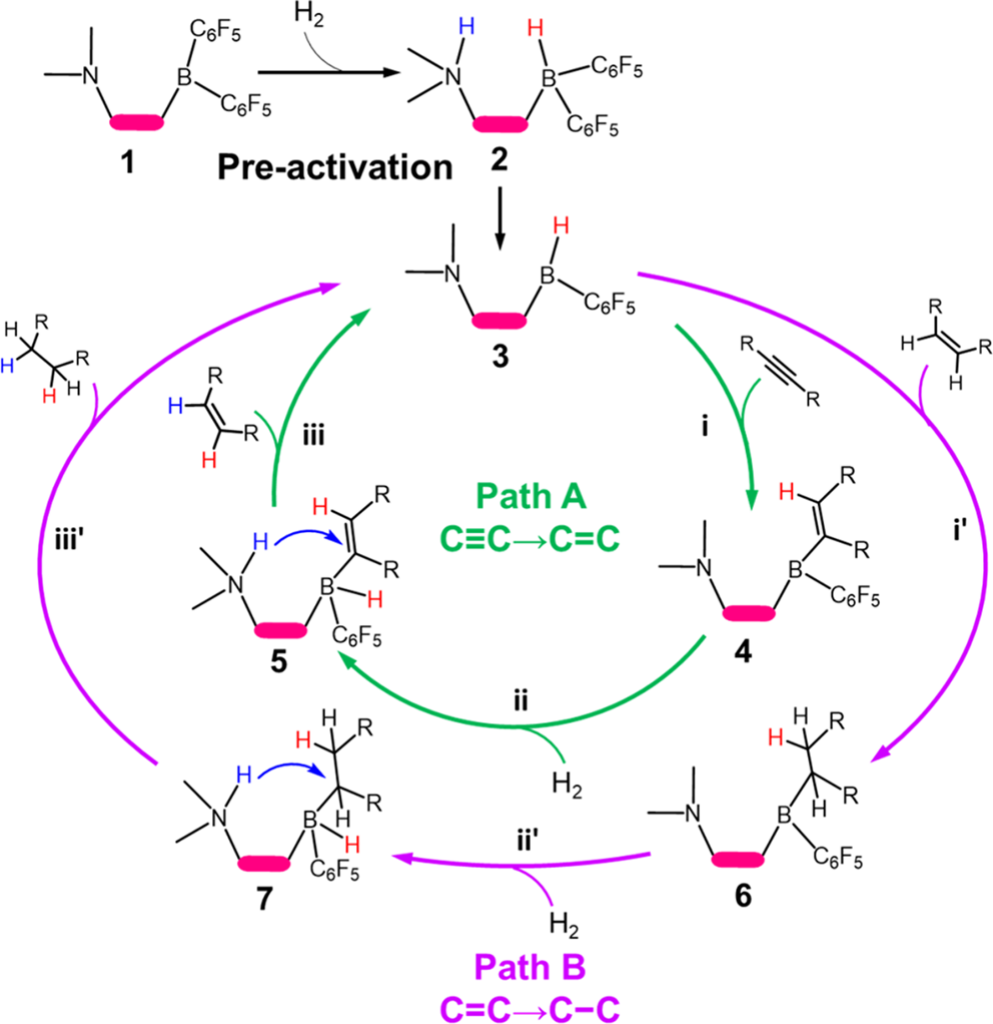
Mechanism of semihydrogenation of alkynes catalyzed by FLPs: precatalyst activation by H_2_ (in black), hydrogenation of alkynes to alkenes (Path A in green) and hydrogenation of alkenes to alkanes (Path B in purple).

The reaction mechanism for alkyne semihydrogenation catalyzed by the FLP is demonstrated in Figure 2. The preactivation of the FLP by H_2_ is required before catalysis, which involves H_2_ splitting to produce a hydride bound at B and a proton bound at N (**1** → **2**), and the intramolecular proton transfer to −C_6_F_5_ leading to the elimination of C_6_F_5_H (**2** → **3**). When the FLP is activated, alkyne hydrogenation proceeds via two possible reaction pathways: Path A involves the hydrogenation of alkynes to alkenes (C≡C → C=C), while Path B involves the hydrogenation of alkenes to alkanes (C=C → C–C). Each pathway consists of three steps:

(i) **alkyne/alkene insertion** into the B–H bond to form a vinyl, **4** (or alkyl, **6**)
(ii) **H**_**2**_**heterolysis** leads to H_2_ splitting and produces **5** (or **7**)
(iii) **intramolecular protonation** via the proton migrates to vinyl (or alkyl) species to produce alkenes (or alkanes) and regenerate the catalyst **3**, and the full catalytic cycle is completed.

To evaluate the activity and selectivity of each FLP catalyst (**a1**–**g1**) toward alkyne semihydrogenation, the energetic span model developed by Kozuch and Shaik^76,77^ was used to predict the overall activation free energies and turnover frequency (TOF). In the energetic span model, kinetics is determined by the relative free energies of all of the intermediates and transition states in the catalytic cycle, rather than by a single step. This model provides an efficient way to calculate the TOF based on free energy profiles, translating the energy data from DFT calculations into the time representation used in experimental reports. The energetic span model yields
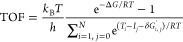
1where *k*_B_ is Boltzmann’s constant, *T* is temperature, *h* is Planck’s constant, *R* is the gas constant, *N* is the number of steps in the catalytic cycle, Δ*G*_r_ is the free energy of the overall catalytic reaction, *T_i_* is the free energy of the transition state *i* (*i* = 1, 2, 3, ···), *I*_*j*_ is the free energy of the intermediate *j* (*j* = 0, 1, 2, 3, ···), and δ*G*_*i*,*j*_^′^ is defined as
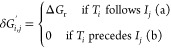
2

For the systems studied here, the approximated energetic span model was used, as shown in eq 3
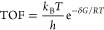
3where the TOF is normally dominated by two states: the TOF-determining transition state (TDTS) and the TOF-determining intermediate (TDI), and δ*G* is defined as
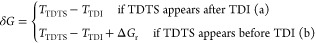
4

**Figure 3:**
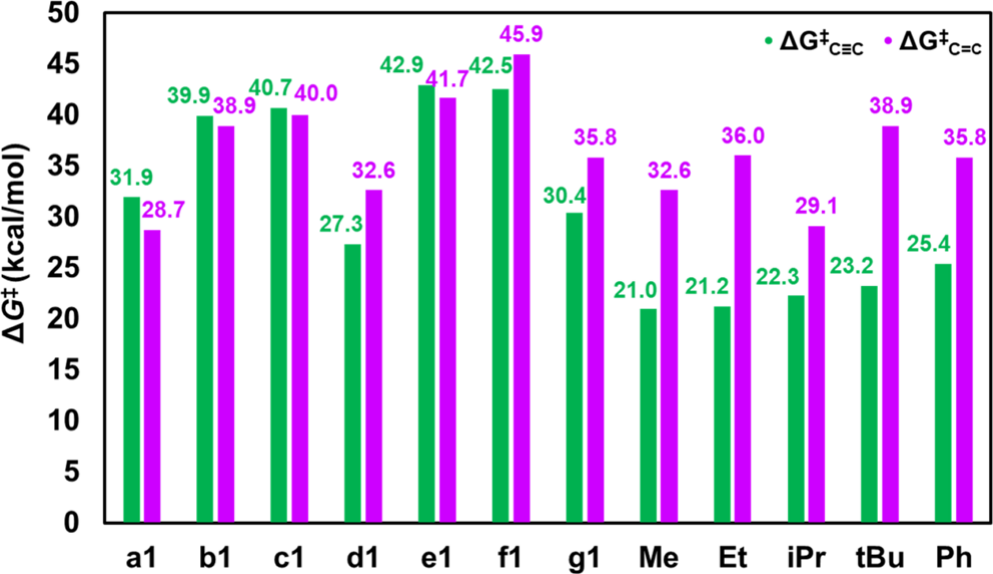
Apparent activation free energies for acetylene hydrogenation to ethene (Δ*G*_C≡C_^⧧^) and ethene hydrogenation to ethane (Δ*G*_C=C_^⧧^) catalyzed by **a1**–**g1** and varied alkyne and alkene hydrogenation catalyzed by **d1**.

The intermediates and transition states were identified for each FLP catalyst (**a1**–**g1**) and the complete set of electronic energies, enthalpies, and Gibbs free energies for all stationary points, as well as Cartesian coordinates of their structures, are provided in the Supporting Information. The free energy profiles for alkyne semihydrogenation catalyzed by FLP catalysts (**a1**–**g1**) are summarized in Figures S1–S12. The apparent activation energies for alkyne hydrogenation to alkenes (Δ*G*_C≡C_^⧧^) and alkene hydrogenation to alkanes (Δ*G*_C=C_^⧧^) are shown in Figure 3 and the TOF data are summarized in Table S1.

When the phenyl linker of **d1** is replaced with linear alkyl (**a**–**c**) and benzyl (**e**), the FLP catalysts (**a1**-**c1**) exhibit higher apparent activation energies for both alkyne and alkene hydrogenation compared to **d1** and higher selectivity toward alkanes over alkenes. Conversely, when the phenyl linker of **d1** is substituted with naphthalenyl (**f**) and dimethyl-xanthenyl (**g**), the FLP catalysts (**f1** and **g1**) also display higher apparent activation energies for both alkyne and alkene hydrogenation compared to **d1**, but both exhibit a higher selectivity toward alkenes over alkanes.

When the substrate is changed from the least steric alkynes, acetylene (H), to more steric alkynes, but-2-yne (Me), hex-3-yne (Et), 2,5-dimethylhex-3-yne (iPr), 2,2,5,5-tetramethylhex-3-yne (*t*Bu), and 1,2-diphenylethyne (Ph), the apparent activation energies for alkyne hydrogenation catalyzed by **d1** (Δ*G*_C≡C_^⧧^: 21.0 kcal/mol (Me), 21.2 kcal/mol (Et), 22.3 kcal/mol (iPr), 23.2 kcal/mol (*t*Bu), 25.4 kcal/mol (Ph)) are lower than those for acetylene hydrogenation (27.3 kcal/mol), while those for alkene hydrogenation are similar or higher than those for ethene hydrogenation.

The energetic span model predicts that **d1** exhibits similar activity toward but-2-yne (Me: Δ*G*_C≡C_^⧧^ = 21.0 kcal/mol) and hex-3-yne (Et: Δ*G*_C≡C_^⧧^ = 21.2 kcal/mol) and lower activity toward 1,2-diphenylethyne (Ph: Δ*G*_C≡C_^⧧^ = 25.4 kcal/mol). This trend aligns with the experimental results that **d1** showed 100% conversion for but-2-yne (7% mol of **d1**) and hex-3-yne (5% mol of **d1**) at the similar reaction condition, while it showed 50% conversion for 1,2-diphenylethyne under the same reaction condition.^58^ The energetic span model predicts a higher apparent activation energy for more sterically hindered alkynes, 2,5-dimethylhex-3-yne (iPr: Δ*G*_C≡C_^⧧^ = 22.3 kcal/mol) and 2,2,5,5-tetramethylhex-3-yne (*t*Bu: Δ*G*_C≡C_^⧧^ = 23.2 kcal/mol) compared to 1,2-diphenylethyne. The computational prediction agrees with the experimental results that the activity of the FLP decreases with respect to more sterically hindered alkynes.

### 3.2 Steric and Electronic Effects of FLPs

To examine the electronic and steric effects on the reactivity of FLPs toward alkyne semihydrogenation, we analyzed the bond length of LA–LB, partial atomic charges of LA and LB, hydride affinity, proton affinity, and the buried volume of FLPs (Figures S13 and S14), and the data are summarized in Tables S2 and S3. The free energies of reaction and activation of steps i–iii are summarized in Tables S4 and S5.

**Figure 4:**
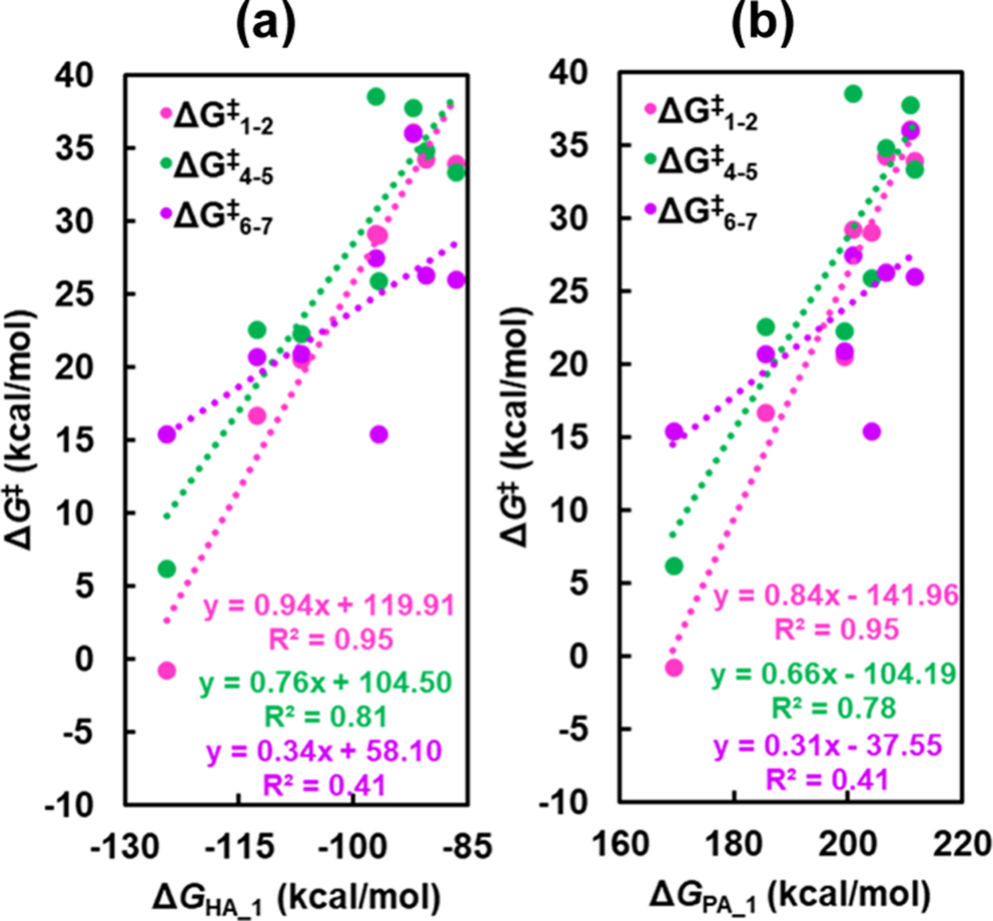
Activation free energies of hydrogen splitting for FLP preactivation (**1** → **2**) and H_2_ heterolysis (**4** → **5** and **6** → **7**) plotted as a function of (a) hydride attachment energies (Δ*G*_HA_1_) and (b) proton attachment energies (Δ*G*_PA_1_).

FLP preactivation (**1** → **2**) and H_2_ heterolysis (**4** → **5** and **6** → **7**) share the same mechanism, involving the heterolytic splitting of H_2_, which yields a hydride bound to B and a proton bound to N. While the difference between these three reactions lies in the different local environment of Lewis acid site, B of **1**, **4** and **6**. For FLPs (**1**), two −C_6_F_5_ groups are bound to B; for **4**, one −C_6_F_5_ group and one vinyl group are bound to B; for **6**, one −C_6_F_5_ group and one alkyl group are bound to B. Therefore, one would expect the factors governing FLP preactivation and H_2_ heterolysis to be similar. The free energies of activation for FLP preactivation (Δ*G*_1–2_^⧧^) and H_2_ heterolysis (Δ*G*_4_^⧧^_–5_ and Δ*G*_6_^⧧^_–7_) are plotted as a function of the hydride attachment energy (Δ*G*_HA_1_) of **a1**–**g1** (Figure 4a) and the proton attachment energy (Δ*G*_PA_1_) of **a1**–**g1** (Figure 4b). Δ*G*_1_^⧧^_–2_ scales with Δ*G*_HA_1_ linearly with an *R*^2^ value of 0.95, indicating an excellent linear correlation and Δ*G*_HA_1_ is a significant predictor of the activation free energies for FLP preactivation. The linear correlation between Δ*G*_4–5_^⧧^ and Δ*G*_HA_1_, with an *R*^2^ value of 0.81, is slightly weaker compared to that between Δ*G*_1–2_^⧧^ and Δ*G*_HA_1_. In contrast, the correlation between Δ*G*_6–7_^⧧^ and Δ*G*_HA_1_ does not follow a linear relationship well, with an *R*^2^ value of 0.41. The declining trend in the *R*^2^ value is attributed to the different chemical environments of the LA site, specifically, the replacement of the electron-withdrawing ligand −C_6_F_5_ of the FLP with the electro-donating ligand vinyl (−CHCH_2_) or alkyl (−CH_2_CH_3_), indicating that the substitution weakens the impact of the hydride attachment energy of the FLP, namely, hydride affinity, on the activation free energy of H_2_ splitting. As shown in Figure 4b, Δ*G*_1–2_^⧧^ also scales linearly with Δ*G*_PA_1_, with an *R*^2^ value of 0.95, suggesting that the proton attachment energy, namely, proton affinity, is as important as hydride affinity for predicting the activation free energies for H_2_ splitting on FLPs. The *R*^2^ value shows a decreasing trend, similar to the trend observed in Figure 4a. Additionally, Δ*G*_1–2_^⧧^ scales linearly with the H_2_ binding free energies at FLPs with the *R*^2^ of 0.94.

**Figure 5:**
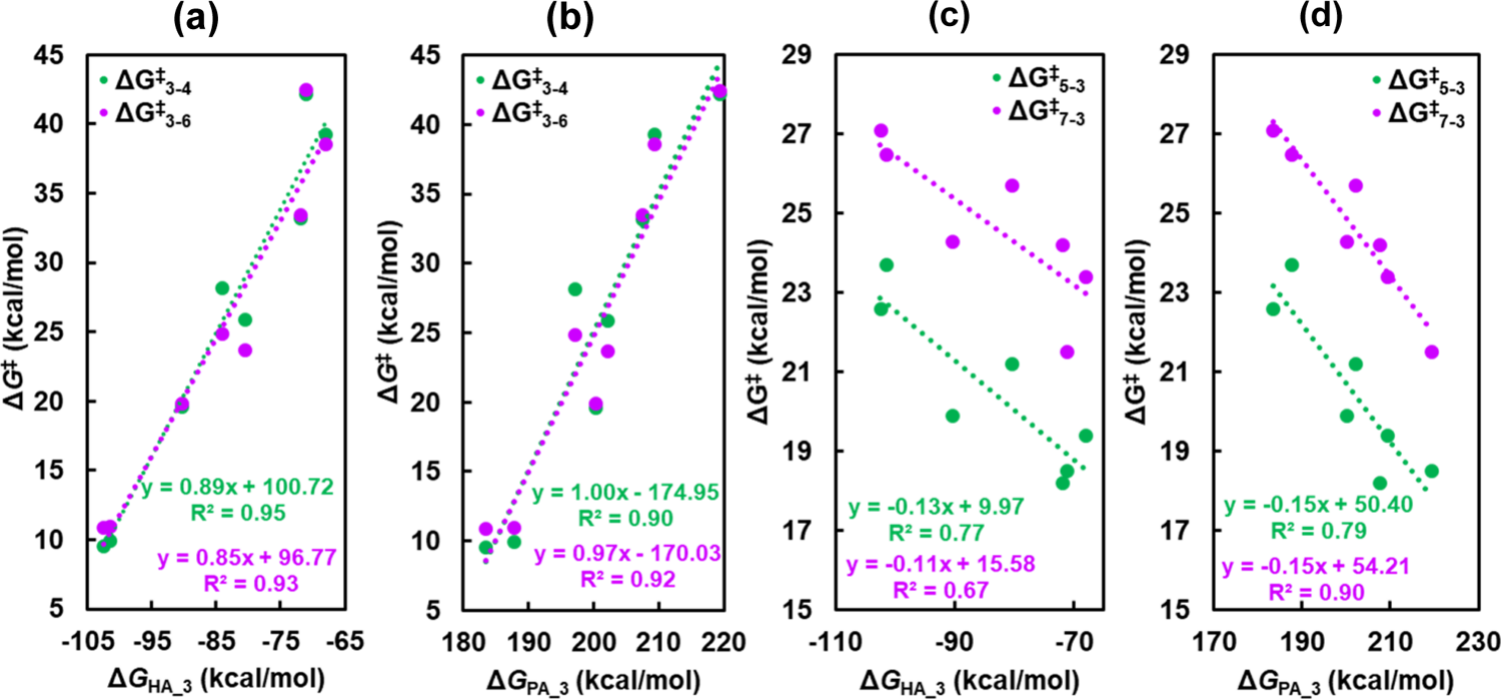
Activation free energies of acetylene/ethene insertion (**3** → **4**/**3** → **6**) and intramolecular protonation (**5** → **3**/**7** → **3**) plotted as a function of (a, c) hydride attachment energies (Δ*G*_HA_3_) and (b, d) proton attachment energies (Δ*G*_PA_3_).

Figure 5a,**b** show the activation free energies of step 1 in acetylene/ethene hydrogenation (**3** → **4**/**3** → **6**), as a function of hydride attachment energies (Δ*G*_HA_3_) and proton attachment energies (Δ*G*_PA_3_) of **a3**–**g3**. The activation free energies of both acetylene and ethene insertion increase as the hydride and proton affinity decreases. The free energies of activation for acetylene insertion (Δ*G*_3–4_^⧧^) and ethene insertion (Δ*G*_3–6_^⧧^) scale linearly with the hydride attachment energies (Δ*G*_HA_3_) of **a3**–**g3** with an *R*^2^ values of 0.95 and 0.93, respectively. Δ*G*_3–4_^⧧^ and Δ*G*_3–6_^⧧^ also scale linearly with the proton attachment energy (Δ*G*_PA_3_) of **a3**–**g3** as well, but with slightly smaller *R*^2^ values (0.90 for Δ*G*_3–4_^⧧^ and 0.92 Δ*G*_3–6_^⧧^). In contrast to acetylene/ethene insertion, the activation free energies of intramolecular protonation (Δ*G*_5–3_^⧧^ and Δ*G*_7–3_^⧧^) decrease with the decrease of the hydride and proton affinity, as shown in Figure 5c,d. This is because the weaker the hydride and proton affinity, the easier is the transfer of the hydride and proton. Δ*G*_5–3_^⧧^ and Δ*G*_7–3_^⧧^ scale linearly with the hydride attachment energy with an *R*^2^ value of 0.77 and 0.67, respectively, and scale linearly with the proton attachment energy with *R*^2^ values of 0.79 and 0.90, respectively. The activation free energies of acetylene/ethene insertion exhibit good linear correlations with proton and hydride attachment energies, but the correlation is slightly stronger for the hydride attachment energies. This is attributed to the direct involvement of hydride in the acetylene/ethene insertion. The correlations between activation free energies of intramolecular protonation and proton attachment energy have a higher *R*^2^ because the proton detachment directly involves the protonation for the release of alkenes or alkanes from FLPs.

**Figure 6:**
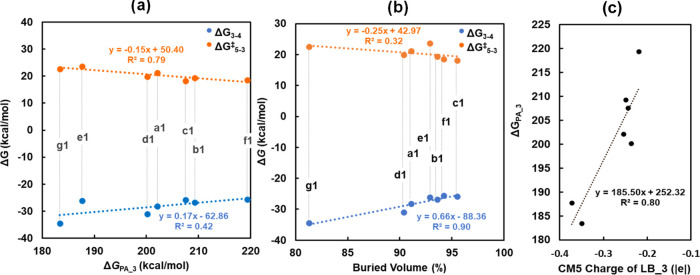
Correlation between the free energies of reaction for alkyne insertion (3 → 4, Δ*G*_3–4_) and the free activation energies for intramolecular protonation (5 → 3, Δ*G*_5–3_^⧧^) catalyzed by **a1**–**g1** and (a) proton attachment energies (Δ*G*_PA_3_) of **a3**–**g3** and (b) the buried volume of FLP catalysts (**a1**–**g1**). (c) Correlation between Δ*G*_PA_3_ and CM5 charge of the Lewis base site (N) of **a3**–**g3**.

The TDI and TDTS are consistent across all FLP catalysts (**a1**–**g1**) for acetylene semihydrogenation. Specifically, for acetylene hydrogenation to ethene, the TDI and TDTS are **4** and **TS**_**5**–**3**_, respectively; for ethene hydrogenation to ethane, the TDI and TDTS are **6** and **TS**_**7**–**3**_, respectively. Therefore, the catalytic activity and selectivity of FLPs for alkyne semihydrogenation could be predicted by identifying the correlations between the energies of the TDTS and TDI and relevant properties or structures. For alkyne hydrogenation to alkene, the TDI and TDTS are **4** and **TS**_**5**–**3**_, respectively. Given the importance of the hydride and proton affinity in predicting the activation free energies for each elementary step in alkyne semihydrogenation, we plotted the free energies of reaction for alkyne insertion (**3** → **4**, Δ*G*_3–4_) and the free energies of activation for intramolecular protonation (**5** → **3**, Δ*G*_5–3_^⧧^) as a function of both hydride and proton attachment energies. As shown in Figure 5c,d, Δ*G*_5–3_^⧧^ scales linearly with the hydride and proton attachment energies with *R*^2^ values of 0.77 and 0.79, respectively. However, Δ*G*_3–4_ does not show a linear relationship between hydride and proton attachment energies, as indicated by the small *R*^2^ values of 0.40 and 0.42, respectively (see Figures 6a and S15a). The substitution of the LA–LB linker (**d**) with **a**–**c** and **e**–**g** alters the distance between the LA and LB sites (*d*_B–N_) and the steric hindrance at the neighboring B and N sites (Table S3). To explore the correlations between the energies of TOF-determining steps and the steric hindrance of FLPs, we plotted Δ*G*_3–4_ and Δ*G*_5–3_^⧧^ as functions of *d*_B–N_ and the buried volume of **a1**–**g1**. We observed that Δ*G*_3–4_ shows a stronger linear correlation with the buried volume of FLPs, with an *R*^2^ of 0.90 (Figure 6b) compared to that of *d*_B–N_, which has an *R*^2^ of 0.69 (Figure S15b). Specifically, the greater the buried volume and thus the stronger the steric hindrance, the less exergonic are the reaction free energies for alkyne insertion. This is due to the steric hindrance weakening the binding of alkynes at the LA site (B). However, Δ*G*_5–3_^⧧^ does not linearly correlate with the buried volume with an *R*^2^ of 0.32, which makes sense because intramolecular protonation involves proton binding at the Lewis base site (N) and subsequent migration to vinyl for alkene production. The steric hindrance of FLPs on the free activation energies (Δ*G*_5–3_^⧧^) of intramolecular protonation could be negligible because the proton is too small. Overall, the free energies of reaction for alkyne insertion (3 → 4, Δ*G*_3–4_) are found to scale linearly with the buried volume of FLPs, and the free activation energies for intramolecular protonation (5 → 3, Δ*G*_5–3_^⧧^) scale linearly with the proton affinity. Furthermore, we observed that proton affinity (Δ*G*_PA_3_) shows a good linear correlation with the CM5 charge of the LB site, N. The activation free energies of acetylene/ethene insertion (**3** → **4**/**3** → **6**) and intramolecular protonation (**5** → **3**/**7** → **3**) as a function of CM5 charge of LA and LB sites are depicted in Figure S16. Overall, a more negative charge on N and the greater electron density at N lead to a stronger proton attachment energy and more exergonic reaction free energies for alkyne insertion. The results suggest that the steric hindrance at the neighboring B and N sites strongly affects the stability of **4**, the alkyne insertion product, while the electron density at the LB site strongly influences the free energy of activation for intramolecular protonation. Varying the LA–LB linkers modifies both steric and electronic properties of FLPs, ultimately affecting their catalytic activity.

Linear correlations were also observed for alkene hydrogenation to alkanes catalyzed by **a1**–**g1**. As shown in Figure S17, the free energies of reaction for alkene insertion (3 → 6, Δ*G*_3–6_) scale linearly with the buried volume of FLP catalysts with an *R*^2^ of 0.64, and the free activation energies for intramolecular protonation (7 → 3, Δ*G*_7–3_^⧧^) scale linearly with the proton affinity with an *R*^2^ of 0.90.

### 3.3 Steric Effect of Alkynes

**Figure 7:**
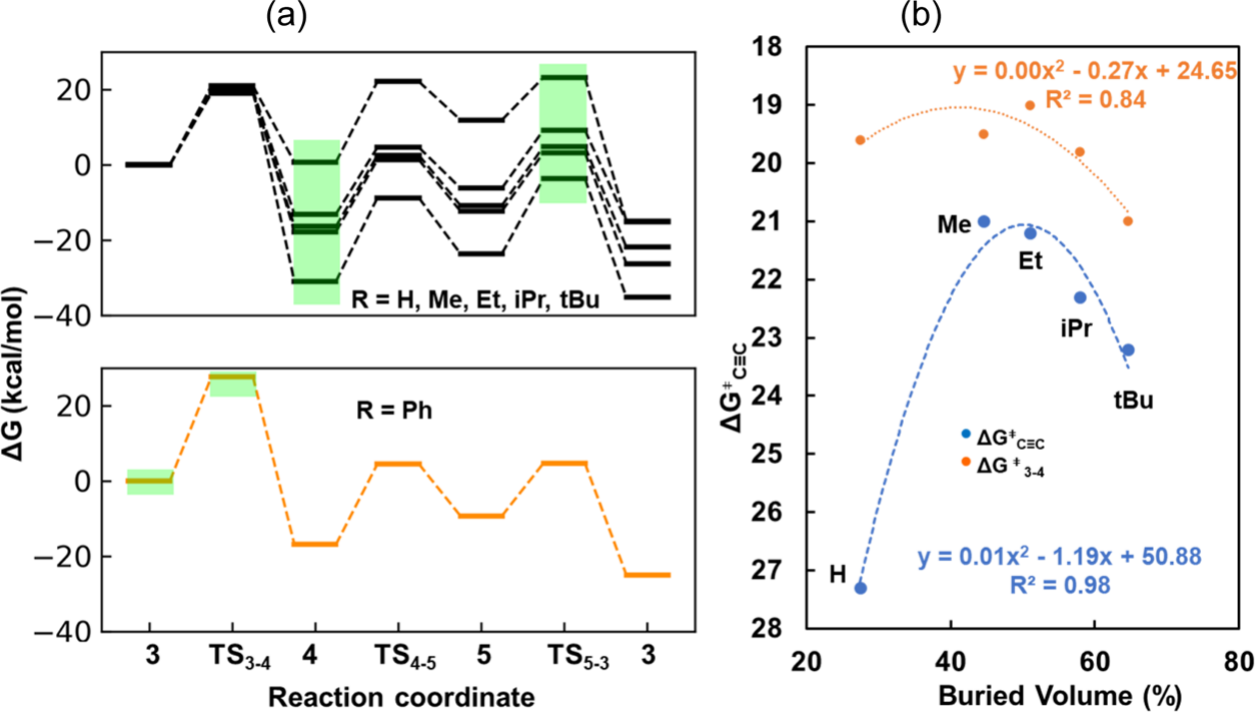
(a) Free energy profiles for alkyne hydrogenation to alkenes catalyzed by **a3**. (b) Correlation between the apparent activation energies (Δ*G*_C≡C_^⧧^) for alkyne hydrogenation and the activation free energies of alkyne insertion (*G*_3–4_^⧧^) and the buried volume of alkynes.

When alkynes with different steric hindrances are catalyzed by the same FLP (**d1**), the TDI and TDTS are not consistent for alkyne hydrogenation to alkenes. As shown in Figure 7a, the TDI and TDTS are **4** and **TS**_**5**–**3**_ for alkyne (H, Me, Et, iPr, and *t*Bu) hydrogenation to alkenes, respectively. For Ph, the TDI and TDTS shift to **3** and **TS**_**3**–**4**_, respectively. Our computational results indicate that for alkynes with a strong steric hindrance (stronger than diphenylacetylene (Ph)), the overall activity for alkyne hydrogenation is primarily determined by the alkyne insertion step, namely, the hydroboration step. In other words, alkyne insertion becomes the rate-limiting step in the overall reaction. This computational discovery aligns with experimental observations where diphenylacetylene remains largely intact with **d3** at room temperature and requires heating to 80 °C, making hydroboration the rate-limiting step in its slow hydrogenation.^58^

To examine the steric effect of alkynes on the reactivity of FLPs, we analyzed the buried volumes of alkynes (excluding Ph, as its TDI and TDTS differ significantly from those of less sterically hindered alkynes; see Table S6). Both Δ*G*_3–4_ and Δ*G*_5–3_^⧧^ show linear correlations with the buried volume of alkynes, with *R*^2^ values of 0.88 and 0.85, respectively (Figure S18a). Specifically, a greater buried volume of alkynes, indicating a stronger steric hindrance, leads to less exergonic reaction free energies for alkyne insertion due to the weakened binding of alkynes at the LA site. Conversely, for intramolecular protonation, a greater buried volume of alkynes results in smaller activation free energies for protonation. This is because higher steric crowding near the LA site hinders tight binding of sterically hindered alkynes and facilitates the dissociation of protonated products (alkenes) from the FLP.^21^ For alkene hydrogenation, the free energies of reaction for alkene insertion (3 → 6, Δ*G*_3–6_) and the free activation energies for intramolecular protonation (7 → 3, Δ*G*_7–3_^⧧^) do not show strong linear correlations with the buried volume of FLP catalysts linearly. As shown in Figure S18b, *R*^2^ values of the two linear correlations are 0.66 and 0.50, respectively.

The experimental results indicate a nonlinear relationship between the steric hindrance of alkynes and the reaction rate. The relative rates of hydroboration at room temperature follow the order of but-2-yne:hex-3-yne/diphenylacetylene = 57:136:no reaction.^58^ Despite but-2-yne having less steric hindrance than hex-3-yne, it exhibits a lower activity. Diphenylacetylene, which has the strongest steric hindrance among the three substrates, shows the lowest activity compared to those of the other two. We plotted the apparent activation energies against the buried volume of alkynes and fitted them with a quadratic function, yielding an *R*^2^ of 0.98 (see Figure 7b). The activity of alkyne hydrogenation exhibits a volcano shape, where too small or too large steric hindrance leads to lower activity, aligning with the experimental trend. We also plotted the activation free energies of alkyne insertion against the buried volume of alkynes, which similarly exhibits a volcano shape (see Figure 7b). Further data will be necessary to fully understand the correlation between activity and steric effects on alkynes with stronger steric hindrance than Ph.

## 4. Conclusions

We applied density functional theory to investigate the steric effect of FLPs and alkynes on the activity toward alkyne semihydrogenation. We modified the steric hindrance of the model catalyst FLP, 1-NMe_2_-2-B(C_6_F_5_)_2_-C_6_H_4_ (**d1**), by replacing the LA-LB linker (**d**) with various linkers **a-c** and **e-g.** The steric effect of alkynes has been investigated by varying the substrate from acetylene (H) to but-2-yne (Me), hex-3-yne (Et), 2,5-dimethylhex-3-yne (iPr), 2,2,5,5-tetramethylhex-3-yne (*t*Bu), and 1,2-diphenylethyne (Ph). For H_2_ splitting on FLPs or FLP-derived intermediates, the activation free energies scale linearly with the hydride and proton affinity of FLPs. For alkyne and alkene hydrogenation, the overall activity is governed by both the electronic and steric effects of FLPs with varied LA-LB linkers. Specifically, the TDI and TDTS energies correlate linearly with the buried volume and proton affinity of FLPs, respectively. Steric crowding at LA sites affects the stability of the alkyne insertion products (**4** and **6**), while the electron density at Lewis base sites influences proton binding energies and thus affects the activation free energy for intramolecular protonation. When different substrates with varied steric hindrances are catalyzed by the same FLP, the activity of alkyne hydrogenation exhibits a volcano shape. Ethyl (Et) is at the peak of the volcano, indicating the highest activity, while alkynes with either smaller or larger steric hindrances than Et show lower activity. Additionally, we observe that for phenyl (Ph), the TDI and TDTS shift to **3** and **TS**_**3**–**4**_. Thus, for alkynes with a greater steric hindrance than Ph, the activity is determined by the rate-determining step, alkyne insertion. To improve the FLP activity for alkyne semihydrogenation, designs should: (1) increase the steric hindrance of the LA neighboring environment to destabilize the alkyne insertion product and raise the energy levels of **4** or **6** and (2) reduce the electron density at the LB site to weaken proton binding, facilitate proton migration, and lower the energy level of **TS**_**5**–**3**_ or **TS**_**7**–**3**_. Therefore, replacing the LA-LB linker with varied steric hindrances and grafting it with electron-withdrawing or electron-donating groups would be a strategy to tune both the steric and electronic properties of FLPs. Additionally, the quantitative structure–activity relationships identified in this work provide valuable guidance for designing FLPs for hydrogenation reactions, extending beyond the scope of alkyne semihydrogenation.

## Supporting Information Available

Electronic energies, enthalpies, and Gibbs free energies of all structures in both the gas phase and benzene (XLSX)
xyz coordinates for all structures (XYZ)
Gibbs free energy data in all figures; Gibbs free energy profiles; correlations; turnover frequencies; and buried volume (PDF)

## Supplementary Material

jp4c05333_si_001.xlsx

jp4c05333_si_002.xyz

## References

[ref1] McCahill J. S. J.; Welch G. C.; Stephan D. W. Reactivity of “Frustrated Lewis Pairs”: Three-Component Reactions of Phosphines, a Borane, and Olefins. Angew. Chem., Int. Ed. 2007, 46, 4968–4971. 10.1002/anie.200701215. 17526043

[ref2] Welch G. C.; Juan R. R. S.; Masuda J. D.; Stephan D. W. Reversible, Metal-Free Hydrogen Activation. Science 2006, 314, 1124–1126. 10.1126/science.1134230. 17110572

[ref3] Stephan D. W. The Broadening Reach of Frustrated Lewis Pair Chemistry. Science 2016, 354, aaf7229 10.1126/science.aaf7229. 27940818

[ref4] Stephan D. W. Frustrated Lewis Pairs. J. Am. Chem. Soc. 2015, 137, 10018–10032. 10.1021/jacs.5b06794. 26214241

[ref5] Stephan D. W. Frustrated Lewis Pairs: From Concept to Catalysis. Acc. Chem. Res. 2015, 48, 306–316. 10.1021/ar500375j. 25535796

[ref6] Stephan D. W. Frustrated Lewis Pair Catalysis: An Introduction; Springer: Cham, 2021; pp 1–28.

[ref7] Stephan D. W. Discovery of Frustrated Lewis Pairs: Intermolecular FLPs for Activation of Small Molecules. In Frustrated Lewis Pairs I; Springer: Berlin, Heidelberg, 2012; pp 1–44. 10.1007/128_2012_381 23208614

[ref8] Stephan D. W.; Erker G. Frustrated Lewis Pair Chemistry of Carbon, Nitrogen and Sulfur Oxides. Chem. Sci. 2014, 5, 2625–2641. 10.1039/C4SC00395K.

[ref9] Soltani Y.; Fontaine F.-G. FLP-Mediated C–H-Activation; Springer: Cham, 2021; pp 113–166.

[ref10] Li N.; Zhang W. X. Frustrated Lewis Pairs: Discovery and Overviews in Catalysis. Chin. J. Chem. 2020, 38, 1360–1370. 10.1002/cjoc.202000027.

[ref11] Stephan D. W. Frustrated Lewis Pairs: A New Strategy to Small Molecule Activation and Hydrogenation Catalysis. Dalton Trans. 2009, 17, 3129–3136. 10.1039/b819621d. 19421613

[ref12] Chen D.; Klankermayer J. Frustrated Lewis Pairs: From Dihydrogen Activation to Asymmetric Catalysis. In Topics in Current Chemistry; Springer, 2013; Vol. 334, pp 1–26. 23408275 10.1007/128_2012_402

[ref13] Stephan D. W. Frustrated Lewis Pair” Hydrogenations. Org. Biomol. Chem. 2012, 10, 5740–5746. 10.1039/c2ob25339a. 22505184

[ref14] Sumerin V.; Chernichenko K.; Schulz F.; Leskelä M.; Rieger B.; Repo T. Amine-Borane Mediated Metal-Free Hydrogen Activation and Catalytic Hydrogenation; Springer: Berlin, Heidelberg, 2012; pp 111–155. 10.1007/128_2012_391 23208615

[ref15] Feng X.; Meng W.; Du H. Frustrated Lewis Pair Catalyzed Asymmetric Reactions; Springer: Cham, 2021; pp 29–86.

[ref16] Wang T.; Daniliuc C. G.; Kehr G.; Erker G. FLP Reduction of Carbon Monoxide and Related Reactions; Springer: Cham, 2021; pp 87–112.

[ref17] Ashley A. E.; O’Hare D. FLP-Mediated Activations and Reductions of CO_2_ and CO. In Topics in Current Chemistry; Springer, 2013; Vol. 334, pp 191–217. 23114497 10.1007/128_2012_377

[ref18] Courtemanche M. A.; Légaré M. A.; Maron L.; Fontaine F. G. Reducing CO_2_ to Methanol Using Frustrated Lewis Pairs: On the Mechanism of Phosphine-Borane-Mediated Hydroboration of CO_2_. J. Am. Chem. Soc. 2014, 136, 10708–10717. 10.1021/ja5047846. 24948159

[ref19] Stephan D. W.; Erker G. Frustrated Lewis Pairs I; Springer: Berlin, Heidelberg, 2013; pp 85–110.

[ref20] Chen J.; Lalancette R. A.; Jäkle F. Chiral Organoborane Lewis Pairs Derived from Pyridylferrocene. Chem. - Eur. J. 2014, 20, 9120–9129. 10.1002/chem.201400057. 24919698

[ref21] Lambic N. S.; Sommer R. D.; Ison E. A. Tuning Catalytic Activity in the Hydrogenation of Unactivated Olefins with Transition-Metal Oxos as the Lewis Base Component of Frustrated Lewis Pairs. ACS Catal. 2017, 7, 1170–1180. 10.1021/acscatal.6b03313.

[ref22] Dorkó É.; Szabó M.; Kótai B.; Pápai I.; Domján A.; Soós T. Expanding the Boundaries of Water-Tolerant Frustrated Lewis Pair Hydrogenation: Enhanced Back Strain in the Lewis Acid Enables the Reductive Amination of Carbonyls. Angew. Chem., Int. Ed. 2017, 56, 9512–9516. 10.1002/anie.201703591. 28591474

[ref23] Mahaut D.; Champagne B.; Berionni G. Frustrated Lewis Pair-Catalyzed Hydrogenation of Unactivated Alkenes with Sterically Hindered 9-Phosphatriptycenes. ChemCatChem 2022, 14, e202200294 10.1002/cctc.202200294.

[ref24] Uhl W.; Wegener P.; Layh M.; Hepp A.; Würthwein E. U. Reaction of an Al/P-Based Frustrated Lewis Pair with Benzophenone: Formation of Adducts and Aluminium Alcoholates via ß-Hydride Elimination. Z. Naturforsch., C 2016, 71, 1043–1050. 10.1515/znb-2016-0118.

[ref25] Normand A. T.; Richard P.; Balan C.; Daniliuc C. G.; Kehr G.; Erker G.; Le Gendre P. Synthetic Endeavors toward Titanium Based Frustrated Lewis Pairs with Controlled Electronic and Steric Properties. Organometallics 2015, 34, 2000–2011. 10.1021/acs.organomet.5b00250.

[ref26] Pla D.; Sadek O.; Cadet S.; Mestre-Voegtlé B.; Gras E. Naphthylaminoborane: From Structural Switches to Frustrated Lewis Pair Reactivity. Dalton Trans. 2015, 44, 18340–18346. 10.1039/C5DT02659H. 26352756

[ref27] Greb L.; Oña-Burgos P.; Schirmer B.; Grimme S.; Stephan D. W.; Paradies J. Metal-Free Catalytic Olefin Hydrogenation: Low-Temperature H_2_ Activation by Frustrated Lewis Pairs. Angew. Chem., Int. Ed. 2012, 51, 10164–10168. 10.1002/anie.201204007. 22936428

[ref28] Zulkifly I.; Protchenko A. V.; Fuentes M. Á.; Hicks J.; Aldridge S. Reactions of a Dimethylxanthene-Derived Frustrated Lewis Pair with Silanes and Stannanes. Z. Anorg. Allg. Chem. 2022, 648, e202200110 10.1002/zaac.202200110.

[ref29] Chase P. A.; Henderson L. D.; Piers W. E.; Parvez M.; Clegg W.; Elsegood M. R. J. Bifunctional Perfluoroaryl Boranes: Synthesis and Coordination Chemistry with Neutral Lewis Base Donors. Organometallics 2006, 25, 349–357. 10.1021/om050764t.

[ref30] Thomas J. M. Handbook Of Heterogeneous Catalysis. 2., Completely Revised and Enlarged Edition. Vol. 1–8. Edited by G. Ertl, H. Knözinger, F. Schüth, and J. Weitkamp. Angew. Chem., Int. Ed. 2009, 48, 3390–3391. 10.1002/anie.200901598.

[ref31] Molnár Á.; Sárkány A.; Varga M. Hydrogenation of Carbon–Carbon Multiple Bonds: Chemo-, Regio- and Stereo-Selectivity. J. Mol. Catal. A: Chem. 2001, 173, 185–221. 10.1016/S1381-1169(01)00150-9.

[ref32] Bonrath W.; Medlock J.; Schütz J.; Wüstenberg B.; Netscher T. Hydrogenation in the Vitamins and Fine Chemicals Industry – An Overview; IntechOpen, 2012.

[ref33] Chen B.; Dingerdissen U.; Krauter J. G. E.; Rotgerink H. G. J. L.; Möbus K.; Ostgard D. J.; Panster P.; Riermeier T. H.; Seebald S.; Tacke T.; Trauthwein H. New Developments in Hydrogenation Catalysis Particularly in Synthesis of Fine and Intermediate Chemicals. Appl. Catal., A 2005, 280, 17–46. 10.1016/j.apcata.2004.08.025.

[ref34] Bonrath W.; Netscher T. Catalytic Processes in Vitamins Synthesis and Production. Appl. Catal., A 2005, 280, 55–73. 10.1016/j.apcata.2004.08.028.

[ref35] Saudan L. A. Hydrogenation Processes in the Synthesis of Perfumery Ingredients. Acc. Chem. Res. 2007, 40, 1309–1319. 10.1021/ar700140m. 17960898

[ref36] Borodziński A.; Bond G. C. Selective Hydrogenation of Ethyne in Ethene-Rich Streams on Palladium Catalysts. Part 1. Effect of Changes to the Catalyst During Reaction. Catal. Rev. 2006, 48, 91–144. 10.1080/01614940500364909.

[ref37] Eggersdorfer M.; Laudert D.; Létinois U.; McClymont T.; Medlock J.; Netscher T.; Bonrath W. One Hundred Years of Vitamins - A Success Story of the Natural Sciences. Angew. Chem., Int. Ed. 2012, 51, 12960–12990. 10.1002/anie.201205886. 23208776

[ref38] Borodziński A.; Bond G. C. Selective Hydrogenation of Ethyne in Ethene-Rich Streams on Palladium Catalysts, Part 2: Steady-State Kinetics and Effects of Palladium Particle Size, Carbon Monoxide, and Promoters. Catal. Rev. 2008, 50, 379–469. 10.1080/01614940802142102.

[ref39] Ertl G.; Knözinger H.; Schüth F.; Weitkamp J. Handbook of Heterogeneous Catalysis; Wiley, 2009; Vol. 48, pp 3390–3391.

[ref40] Galvis H. M. T.; de Jong K. P. Catalysts for Production of Lower Olefins from Synthesis Gas: A Review. ACS Catal. 2013, 3, 2130–2149. 10.1021/cs4003436.

[ref41] Armbrüster M.; Kovnir K.; Friedrich M.; Teschner D.; Wowsnick G.; Hahne M.; Gille P.; Szentmiklósi L.; Feuerbacher M.; Heggen M.; et al. Al_13_Fe_4_ as a Low-Cost Alternative for Palladium in Heterogeneous Hydrogenation. Nat. Mater. 2012, 11, 690–693. 10.1038/nmat3347. 22683821

[ref42] Zacharopoulou V.; Lemonidou A. A. Olefins from Biomass Intermediates: A Review. Catalysts 2018, 8, 2 10.3390/catal8010002.

[ref43] Fakhroleslam M.; Sadrameli S. M. Thermal Cracking of Hydrocarbons for the Production of Light Olefins; A Review on Optimal Process Design, Operation, and Control. Ind. Eng. Chem. Res. 2020, 59, 12288–12303. 10.1021/acs.iecr.0c00923.

[ref44] Schbib N. S.; García M. A.; Gígola C. E.; Errazu A. F. Kinetics of Front-End Acetylene Hydrogenation in Ethylene Production. Ind. Eng. Chem. Res. 1996, 35, 1496–1505. 10.1021/ie950600k.

[ref45] Nikolaev S. A.; Zanaveskin L. N.; Smirnov V. V.; Averyanov V. A.; Zanaveskin K. L. Catalytic Hydrogenation of Alkyne and Alkadiene Impurities from Alkenes. Practical and Theoretical Aspects. Russ. Chem. Rev. 2009, 78, 231–247. 10.1070/RC2009v078n03ABEH003893.

[ref46] Huang L.; Subramanian R.; Wang J.; Oh J. K.; Ye Z. Ligand Screening for Palladium Nanocatalysts towards Selective Hydrogenation of Alkynes. Mol. Catal. 2020, 488, 110923 10.1016/j.mcat.2020.110923.

[ref47] Wang Z.; Garg A.; Wang L.; He H.; Dasgupta A.; Zanchet D.; Janik M. J.; Rioux R. M.; Román-Leshkov Y. Enhancement of Alkyne Semi-Hydrogenation Selectivity by Electronic Modification of Platinum. ACS Catal. 2020, 10, 6763–6770. 10.1021/acscatal.9b04070.

[ref48] Song L.; Feng Q.; Wang Y.; Ding S.; Wu Y.-D.; Zhang X.; Chung L. W.; Sun J. Ru-Catalyzed Migratory Geminal Semihydrogenation of Internal Alkynes to Terminal Olefins. J. Am. Chem. Soc. 2019, 141, 17441–17451. 10.1021/jacs.9b09658. 31596081

[ref49] Desai S. P.; Ye J.; Zheng J.; Ferrandon M. S.; Webber T. E.; Platero-Prats A. E.; Duan J.; Garcia-Holley P.; Camaioni D. M.; Chapman K. W.; et al. Well-Defined Rhodium–Gallium Catalytic Sites in a Metal–Organic Framework: Promoter-Controlled Selectivity in Alkyne Semihydrogenation to E-Alkenes. J. Am. Chem. Soc. 2018, 140, 15309–15318. 10.1021/jacs.8b08550. 30352506

[ref50] Sárkány A.; Geszti O.; Sáfrán G. Preparation of Pdshell–Aucore/SiO2 Catalyst and Catalytic Activity for Acetylene Hydrogenation. Appl. Catal., A 2008, 350, 157–163. 10.1016/j.apcata.2008.08.012.

[ref51] Zheng Y.; Tan T.; Wang C. Seed-Mediated Growth of Alloyed Ag-Pd Shells toward Alkyne Semi-Hydrogenation Reactions under Mild Conditions†. Chin. J. Chem. 2021, 39, 3071–3078. 10.1002/cjoc.202100308.

[ref52] Liu Y.; Liu X.; Feng Q.; He D.; Zhang L.; Lian C.; Shen R.; Zhao G.; Ji Y.; Wang D.; et al. Intermetallic NixMy (M = Ga and Sn) Nanocrystals: A Non-Precious Metal Catalyst for Semi-Hydrogenation of Alkynes. Adv. Mater. 2016, 28, 4747–4754. 10.1002/adma.201600603. 27074143

[ref53] Furukawa S.; Yokoyama A.; Komatsu T. Efficient Catalytic System for Synthesis of Trans -Stilbene from Diphenylacetylene Using Rh-Based Intermetallic Compounds. ACS Catal. 2014, 4, 3581–3585. 10.1021/cs500947d.

[ref54] Furukawa S.; Komatsu T. Selective Hydrogenation of Functionalized Alkynes to (E)-Alkenes, Using Ordered Alloys as Catalysts. ACS Catal. 2016, 6, 2121–2125. 10.1021/acscatal.5b02953.

[ref55] Mömming C. M.; Frömel S.; Kehr G.; Fröhlich R.; Grimme S.; Erker G. Reactions of an Intramolecular Frustrated Lewis Pair with Unsaturated Substrates: Evidence for a Concerted Olefin Addition Reaction. J. Am. Chem. Soc. 2009, 131, 12280–12289. 10.1021/ja903511s. 19658420

[ref56] Jiang C.; Blacque O.; Berke H. Activation of Terminal Alkynes by Frustrated Lewis Pairs. Organometallics 2010, 29, 125–133. 10.1021/om9008636.

[ref57] Voss T.; Mahdi T.; Otten E.; Fröhlich R.; Kehr G.; Stephan D. W.; Erker G. Frustrated Lewis Pair Behavior of Intermolecular Amine/B(C_6_F_5_)_3_ Pairs. Organometallics 2012, 31, 2367–2378. 10.1021/om300017u.

[ref58] Chernichenko K.; Madarász Á.; Pápai I.; Nieger M.; Leskelä M.; Repo T. A Frustrated-Lewis-Pair Approach to Catalytic Reduction of Alkynes to Cis-Alkenes. Nat. Chem. 2013, 5, 718–723. 10.1038/nchem.1693. 23881505

[ref59] Ye J.; McEwen M. Understanding the Reactivity, Selectivity and Deactivation of Frustrated Lewis Pairs for Semihydrogenation of Acetylene. J. Phys. Chem. C 2022, 126, 18605–18616. 10.1021/acs.jpcc.2c05912.

[ref60] Sumerin V.; Schulz F.; Atsumi M.; Wang C.; Nieger M.; Leskelä M.; Repo T.; Pyykkö P.; Rieger B. Molecular Tweezers for Hydrogen: Synthesis, Characterization, and Reactivity. J. Am. Chem. Soc. 2008, 130, 14117–14119. 10.1021/ja806627s. 18826306

[ref61] Schwendemann S.; Fröhlich R.; Kehr G.; Erker G. Intramolecular Frustrated N/B Lewis Pairs by Enamine Hydroboration. Chem. Sci. 2011, 2, 1842–1849. 10.1039/c1sc00124h.

[ref62] Schwendemann S.; Oishi S.; Saito S.; Fröhlich R.; Kehr G.; Erker G. Reaction of an “Invisible” Frustrated N/B Lewis Pair with Dihydrogen. Chem. - Asian J. 2013, 8, 212–217. 10.1002/asia.201200776. 23109376

[ref63] Özgün T.; Ye K. Y.; Daniliuc C. G.; Wibbeling B.; Liu L.; Grimme S.; Kehr G.; Erker G. Why Does the Intramolecular Trimethylene-Bridged Frustrated Lewis Pair Mes_2_PCH_2_CH_2_CH_2_B(C_6_F_5_)_2_ Not Activate Dihydrogen?. Chem. - Eur. J. 2016, 22, 5988–5995. 10.1002/chem.201505200. 26999779

[ref64] Spies P.; Kehr G.; Bergander K.; Wibbeling B.; Fröhlich R.; Erker G. Metal-Free Dihydrogen Activation Chemistry: Structural and Dynamic Features of Intramolecular P/B Pairs. Dalton Trans. 2009, 9, 1534–1541. 10.1039/b815832k. 19421595

[ref65] Li Y. F.; Kang Y.; Ko S. B.; Rao Y.; Sauriol F.; Wang S. Highly Congested Donor-Acceptor P-B Compound: Synthesis and Properties of a BMes_2_- and a PPh_2_-Functionalized 1,8-Naphthalene. Organometallics 2013, 32, 3063–3068. 10.1021/om4002846.

[ref66] Frisch M. J.; Trucks G. W.; Schlegel H. B.; Scuseria G. E.; Robb M. A.; Cheeseman J. R.; Scalmani G.; Barone V.; Mennucci B.; Petersson G. A.; Nakatsuji H.; Caricato M.; Li X.; Hratchian H. P.; Izmaylov A. F.; Bloino J.; Zheng G.; Sonnenberg J. L.; Hada M.; Ehara M.; Toyota K.; Fukuda R.; Hasegawa J.; Ishida M.; Nakajima T.; Honda Y.; Kitao O.; Nakai H.; Vreven T.; Montgomery J. A. Jr.; Peralta J. E.; Ogliaro F.; Bearpark M.; Heyd J. J.; Brothers E.; Kudin K. N.; Staroverov V. N.; Kobayashi R.; Normand J.; Raghavachari K.; Rendell A.; Burant J. C.; Iyengar S. S.; Tomasi J.; Cossi M.; Rega N.; Millam J. M.; Klene M.; Knox J. E.; Cross J. B.; Bakken V.; Adamo C.; Jaramillo J.; Gomperts R.; Stratmann R. E.; Yazyev O.; Austin A. J.; Cammi R.; Pomelli C.; Ochterski J. W.; Martin R. L.; Morokuma K.; Zakrzewski V. G.; Voth G. A.; Salvador P.; Dannenberg J. J.; Dapprich S.; Daniels A. D.; Farkas Ö.; Foresman J. B.; Ortiz J. V.; Cioslowski J.; Fox D. J. Gaussian 16, revision 01C; Gaussian, Inc.: Wallingford, CT, 2016.

[ref67] Zhao Y.; Truhlar D. G. The M06 Suite of Density Functionals for Main Group Thermochemistry, Thermochemical Kinetics, Noncovalent Interactions, Excited States, and Transition Elements: Two New Functionals and Systematic Testing of Four M06-Class Functionals and 12 Other Function. Theor. Chem. Acc. 2008, 120, 215–241. 10.1007/s00214-007-0310-x.

[ref68] Weigend F. Accurate Coulomb-Fitting Basis Sets for H to Rn. Phys. Chem. Chem. Phys. 2006, 8, 1057–1065. 10.1039/b515623h. 16633586

[ref69] Weigend F.; Ahlrichs R. Balanced Basis Sets of Split Valence, Triple Zeta Valence and Quadruple Zeta Valence Quality for H to Rn: Design and Assessment of Accuracy. Phys. Chem. Chem. Phys. 2005, 7, 3297–3305. 10.1039/b508541a. 16240044

[ref70] Marenich A. V.; Cramer C. J.; Truhlar D. G. Universal Solvation Model Based on Solute Electron Density and on a Continuum Model of the Solvent Defined by the Bulk Dielectric Constant and Atomic Surface Tensions. J. Phys. Chem. B 2009, 113, 6378–6396. 10.1021/jp810292n. 19366259

[ref71] Marenich A. V.; Jerome S. V.; Cramer C. J.; Truhlar D. G. Charge Model 5: An Extension of Hirshfeld Population Analysis for the Accurate Description of Molecular Interactions in Gaseous and Condensed Phases. J. Chem. Theory Comput. 2012, 8, 527–541. 10.1021/ct200866d. 26596602

[ref72] Ingman V. M.; Schaefer A. J.; Andreola L. R.; Wheeler S. E. QChASM: Quantum Chemistry Automation and Structure Manipulation. WIREs Comput. Mol. Sci. 2021, 11, e1510 10.1002/wcms.1510.

[ref73] Schaefer A. J.; Ingman V. M.; Wheeler S. E. SEQCROW: A ChimeraX Bundle to Facilitate Quantum Chemical Applications to Complex Molecular Systems. J. Comput. Chem. 2021, 42, 1750–1754. 10.1002/jcc.26700. 34109660

[ref74] Meng E. C.; Goddard T. D.; Pettersen E. F.; Couch G. S.; Pearson Z. J.; Morris J. H.; Ferrin T. E. UCSF ChimeraX: Tools for structure building and analysis. Protein Sci. 2023, 32 (11), e4792 10.1002/pro.4792. 37774136 PMC10588335

[ref75] Mantina M.; Chamberlin A. C.; Valero R.; Cramer C. J.; Truhlar D. G. Consistent van Der Waals Radü for the Whole Main Group. J. Phys. Chem. A 2009, 113, 5806–5812. 10.1021/jp8111556. 19382751 PMC3658832

[ref76] Kozuch S.; Shaik S. A Combined Kinetic–Quantum Mechanical Model for Assessment of Catalytic Cycles: Application to Cross-Coupling and Heck Reactions. J. Am. Chem. Soc. 2006, 128, 3355–3365. 10.1021/ja0559146. 16522117

[ref77] Kozuch S.; Shaik S. How to Conceptualize Catalytic Cycles? The Energetic Span Model. Acc. Chem. Res. 2011, 44, 101–110. 10.1021/ar1000956. 21067215

