# Supplementary material for: Theoretical Investigation of the Steric Effects on Alkyne Semihydrogenation Catalyzed by Frustrated Lewis Pairs
Source: J Phys Chem C Nanomater Interfaces. 2024 Nov 7;128(46):19510–8. doi: 10.1021/acs.jpcc.4c05333 (PMC11587091; doi:10.1021/acs.jpcc.4c05333)
Supplement: Supplementary file 3 — jp4c05333_si_003.pdf [file jp4c05333_si_003.pdf]

Supporting Information

Theoretical Investigation of the Steric Effects on Alkyne Semi-hydrogenation Catalyzed by Frustrated Lewis Pairs

Allison Zeiss, Jacob Hartman, Sara Kelemen, Jingyun Ye*

Department of Chemistry and Biochemistry, Duquesne University, Pittsburgh, Pennsylvania 15282, USA

Corresponding author:

Jingyun Ye: yej1@duq.edu

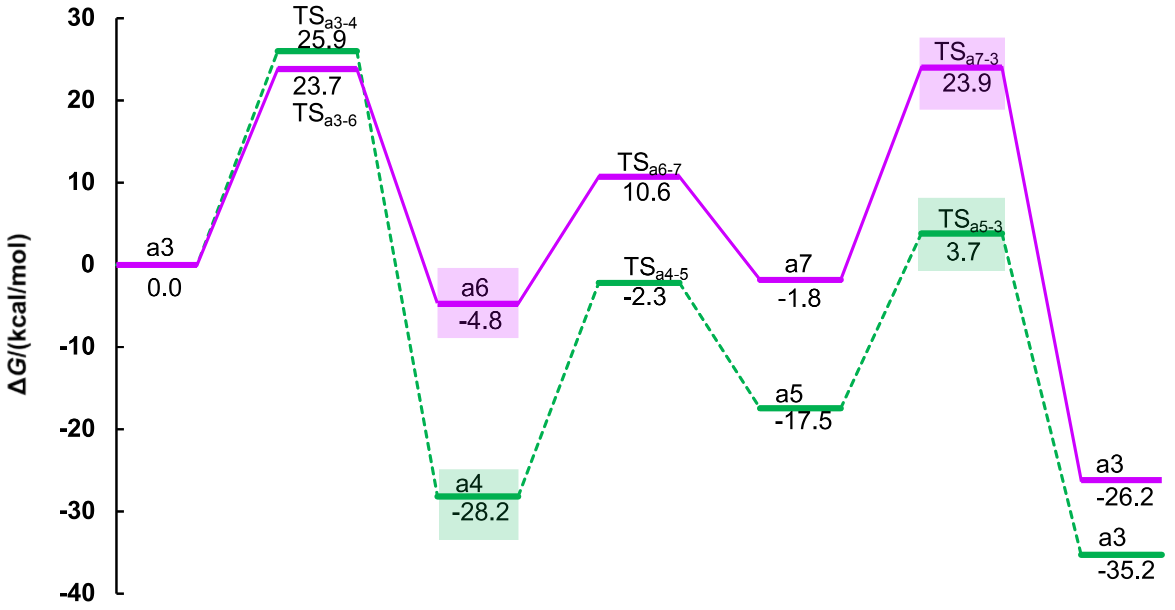

Figure S1. The free energy profile for the hydrogenation of acetylene to ethene (green line) and ethene to ethane (purple line) that catalyzed by **a3**. The TOF-determining intermediate (TDI) and TOF-determining transition state (TDTS) are highlighted in the green or purple boxes along each pathway.

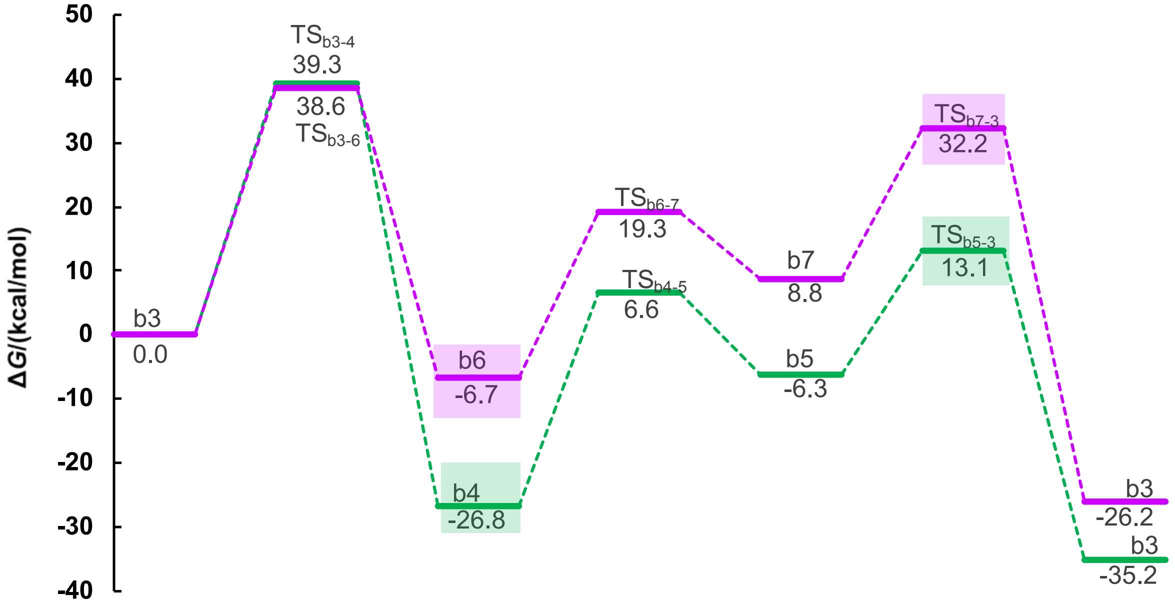

Figure S2. The free energy profile for the hydrogenation of acetylene to ethene (green line) and ethene to ethane (purple line) that catalyzed by **b3**. The TDI and TDTS are highlighted in the green or purple boxes along each pathway.

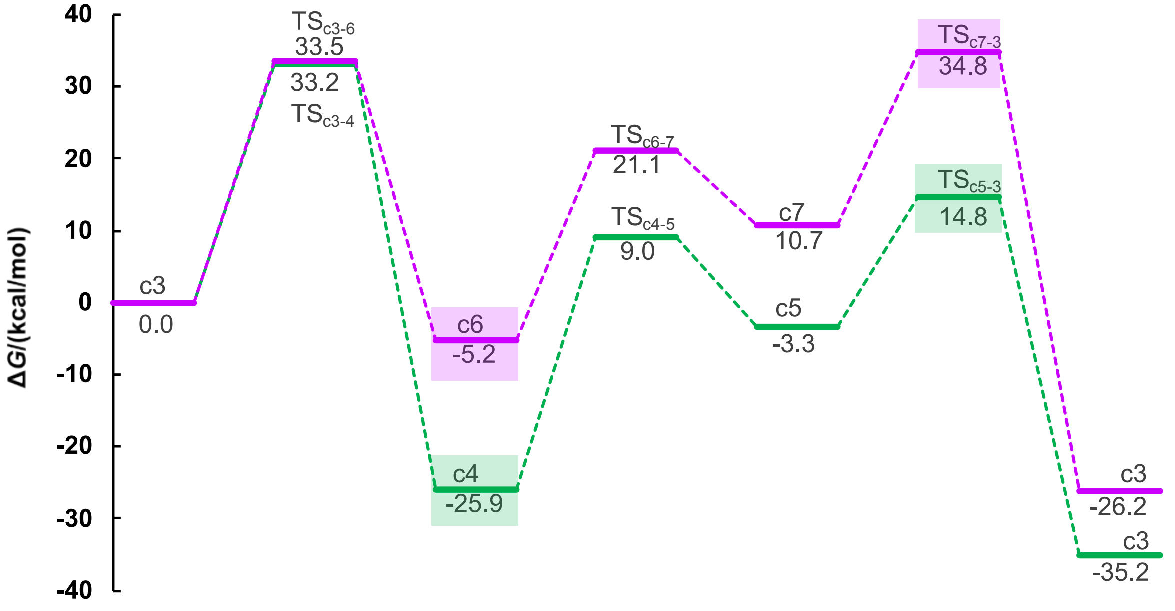

Figure S3. The free energy profile for the hydrogenation of acetylene to ethene (green line) and ethene to ethane (purple line) that catalyzed by **c3**. The TDI and TDTS are highlighted in the green or purple boxes along each pathway.

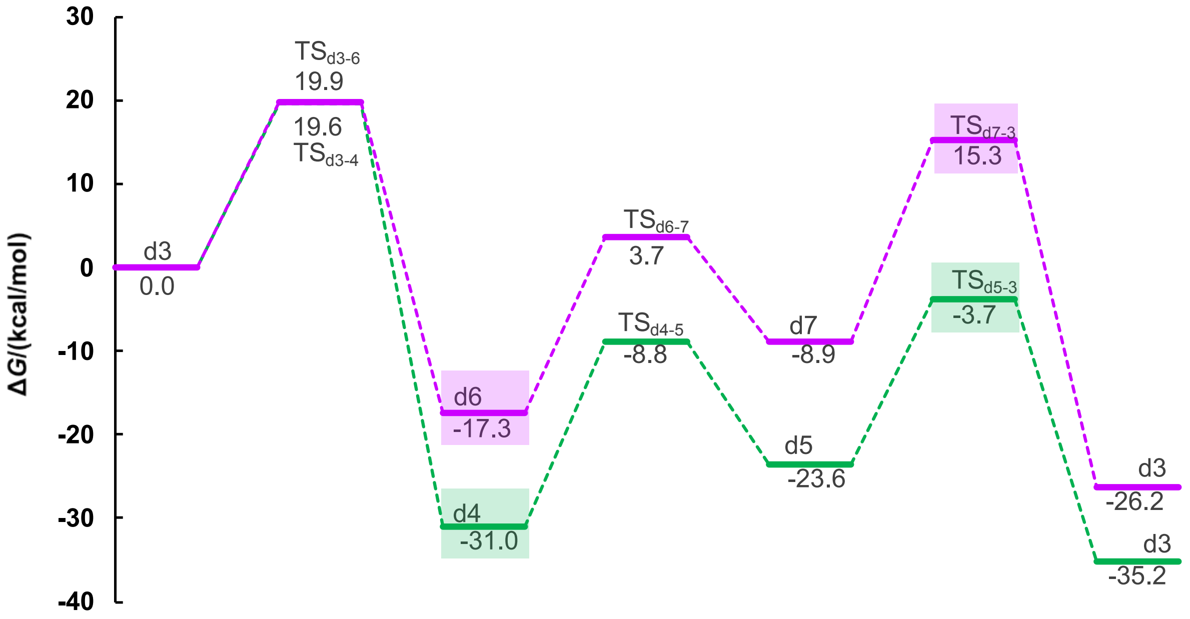

Figure S4. The free energy profile for the hydrogenation of acetylene to ethene (green line) and ethene to ethane (purple line) that catalyzed by **d3**. The TDI and TDTS are highlighted in the green or purple boxes along each pathway.

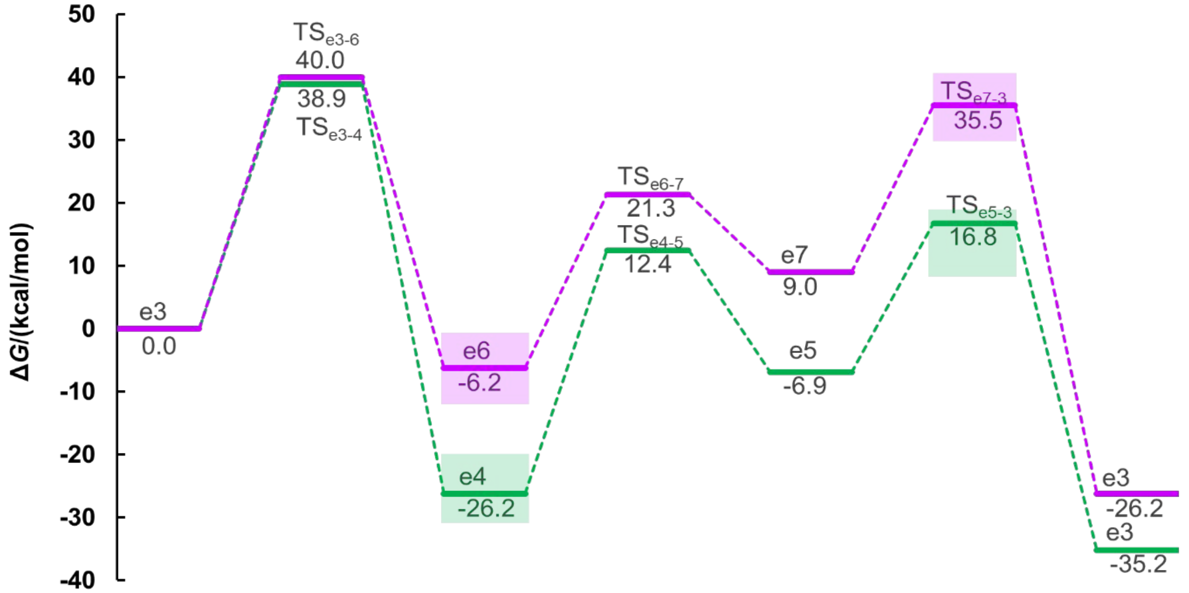

Figure S5. The free energy profile for the hydrogenation of acetylene to ethene (green line) and ethene to ethane (purple line) that catalyzed by **e3**. The TDI and TDTS are highlighted in the green or purple boxes along each pathway.

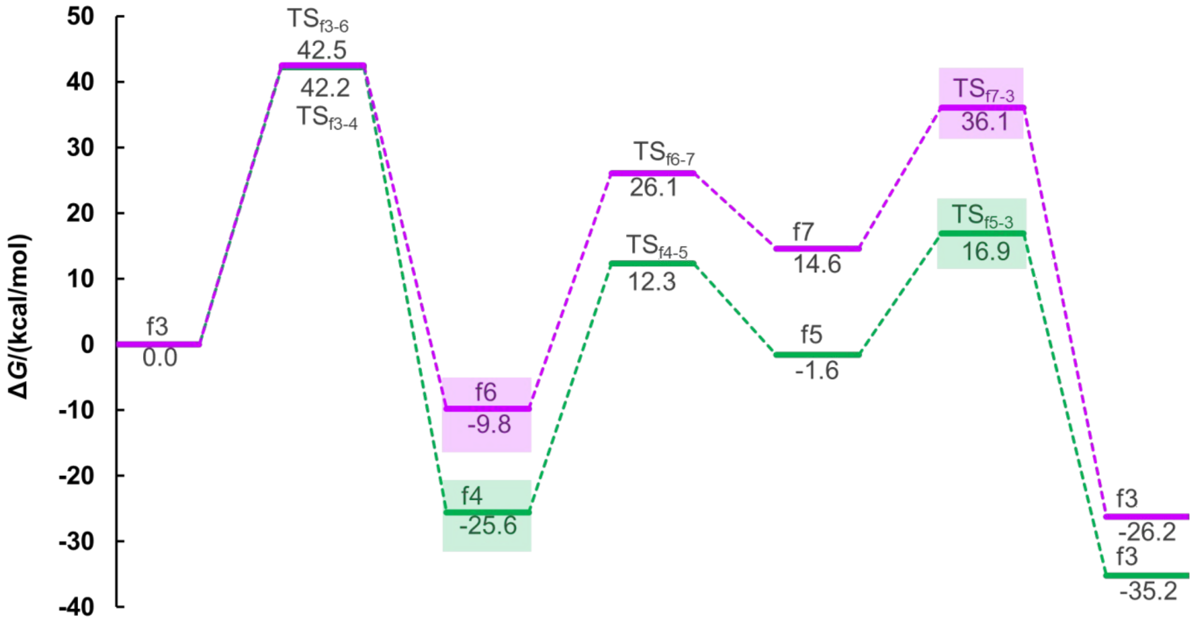

Figure S6. The free energy profile for the hydrogenation of acetylene to ethene (green line) and ethene to ethane (purple line) that catalyzed by **f3**. The TDI and TDTS are highlighted in the green or purple boxes along each pathway.

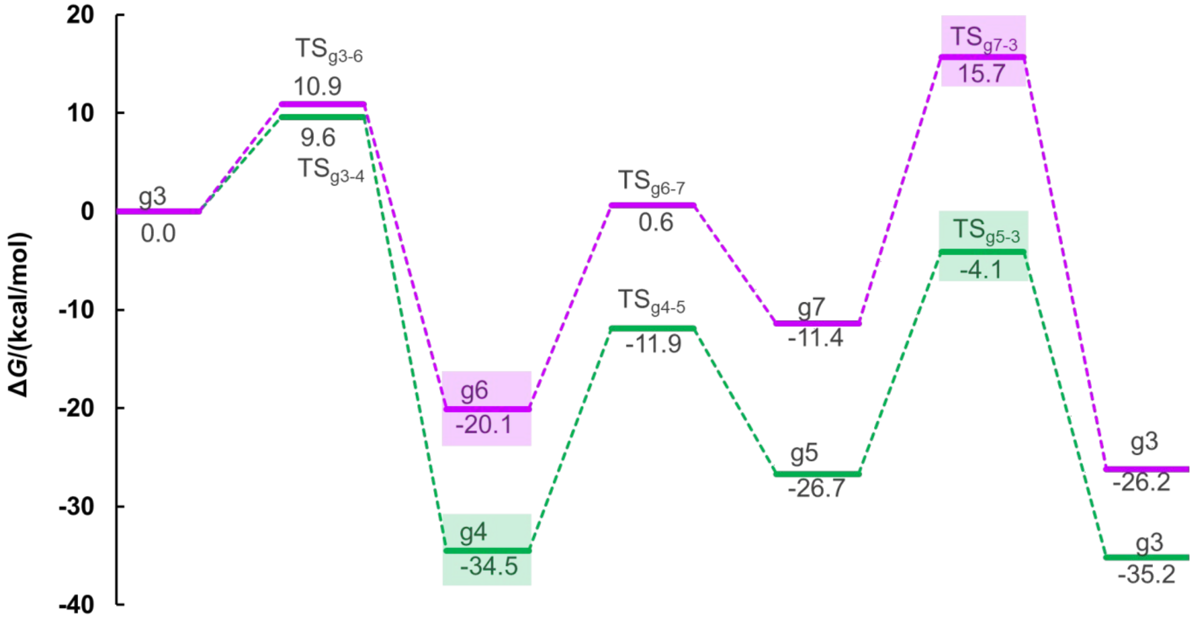

Figure S7. The free energy profile for the hydrogenation of acetylene to ethene (green line) and ethene to ethane (purple line) that catalyzed by **g3**. The TDI and TDTS are highlighted in the green or purple boxes along each pathway.

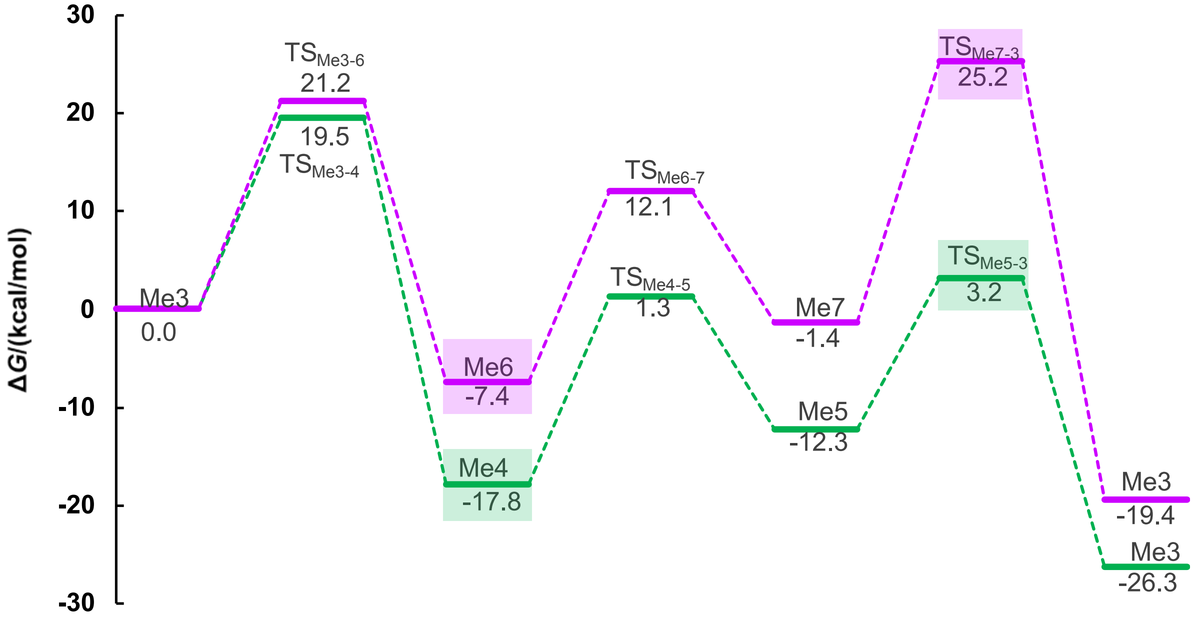

Figure S8. The free energy profile for the hydrogenation of 2-butyne to 2-butene (green line) and 2-butene to 2-butane (purple line) that catalyzed by **a3**. The TDI and TDTS are highlighted in the green or purple boxes along each pathway.

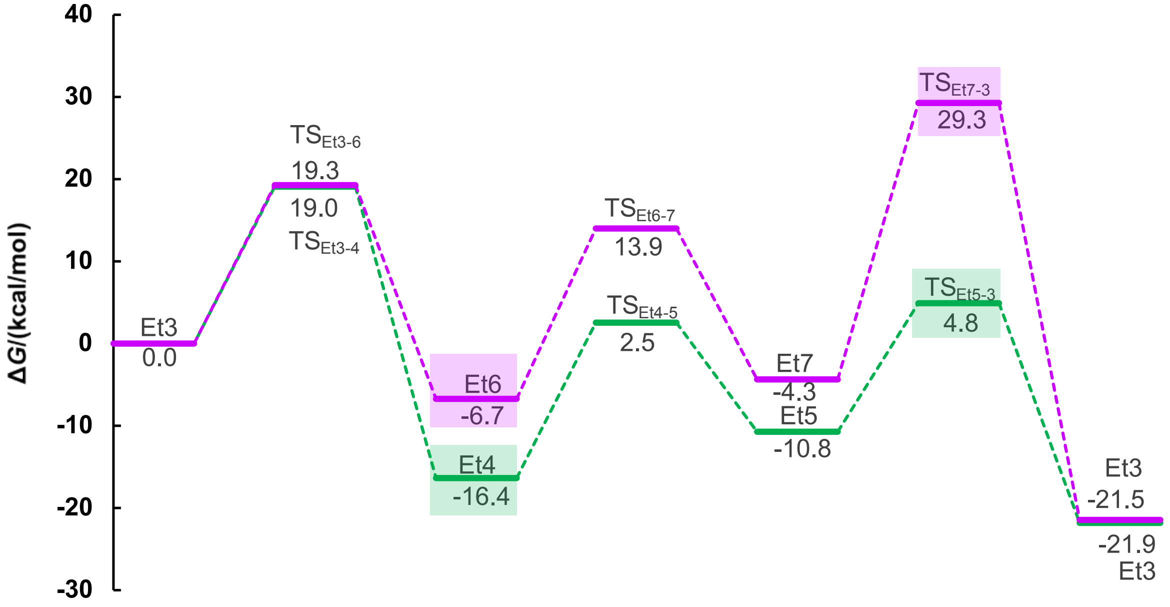

Figure S9. The free energy profile for the hydrogenation of 3-hexyne to 3-hexene (green line) and 3-hexene to hexane (purple line) that catalyzed by **a3**. The TDI and TDTS are highlighted in the green or purple boxes along each pathway.

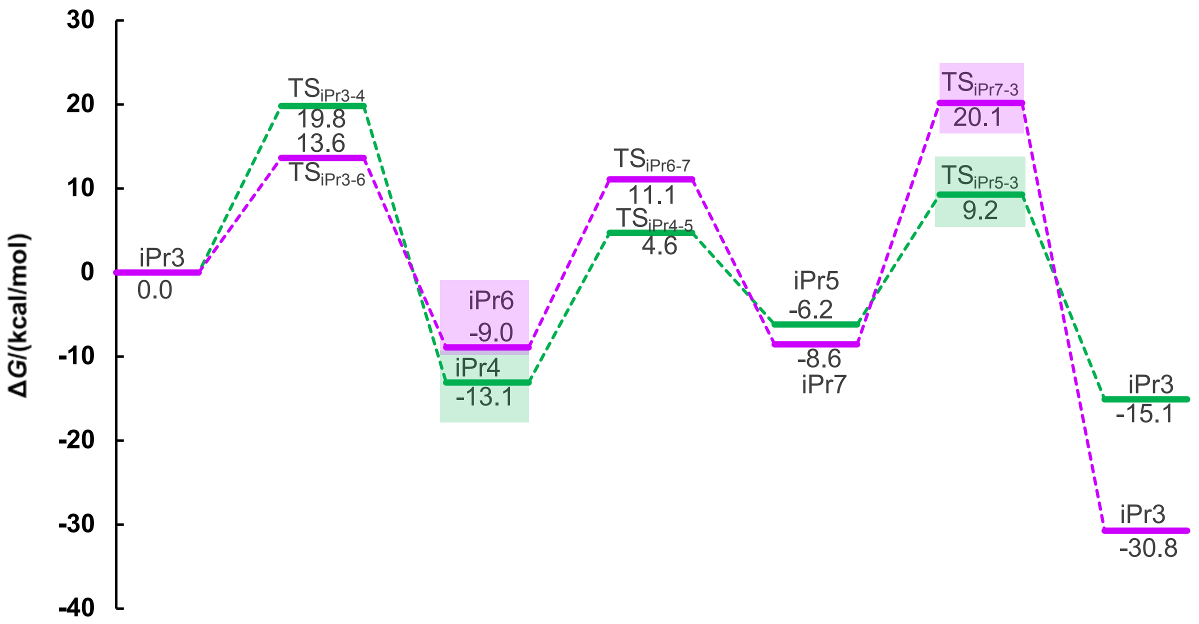

Figure S10. The free energy profile for the hydrogenation of 2,5-dimethylhex-3-yne to 2,5-dimethylhex-3-ene (green line) and 2,5-dimethylhex-3-ene to 2,5-dimethylhexane (purple line) that catalyzed by **a3**. The TDI and TDTS are highlighted in the green or purple boxes along each pathway.

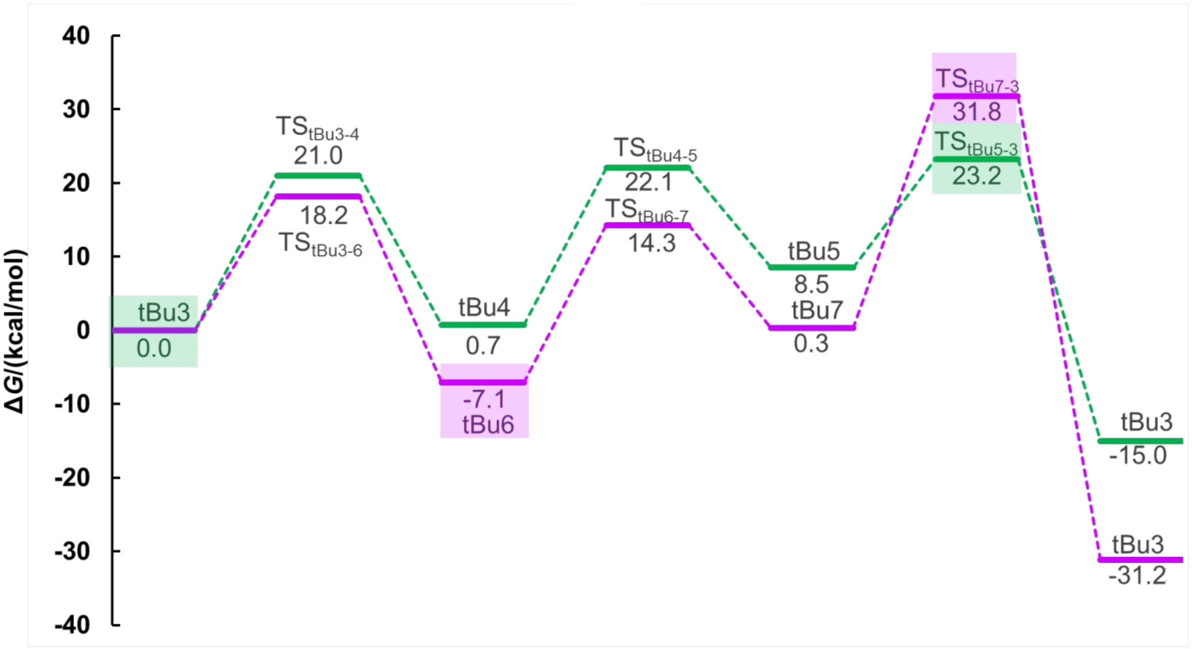

Figure S11. The free energy profile for the hydrogenation of 2,2,5,5-tetramethylhex-3-yne to 2,2,5,5-tetramethylhexene (green line) and 2,2,5,5-tetramethylhexene to 2,2,5,5-tetramethylhexane (purple line) that catalyzed by **a3**. The TDI and TDTS are highlighted in the green or purple boxes along each pathway. (Note: We consider that TDI is either **3 or 4**, because both intermediates are at similar energy level.)

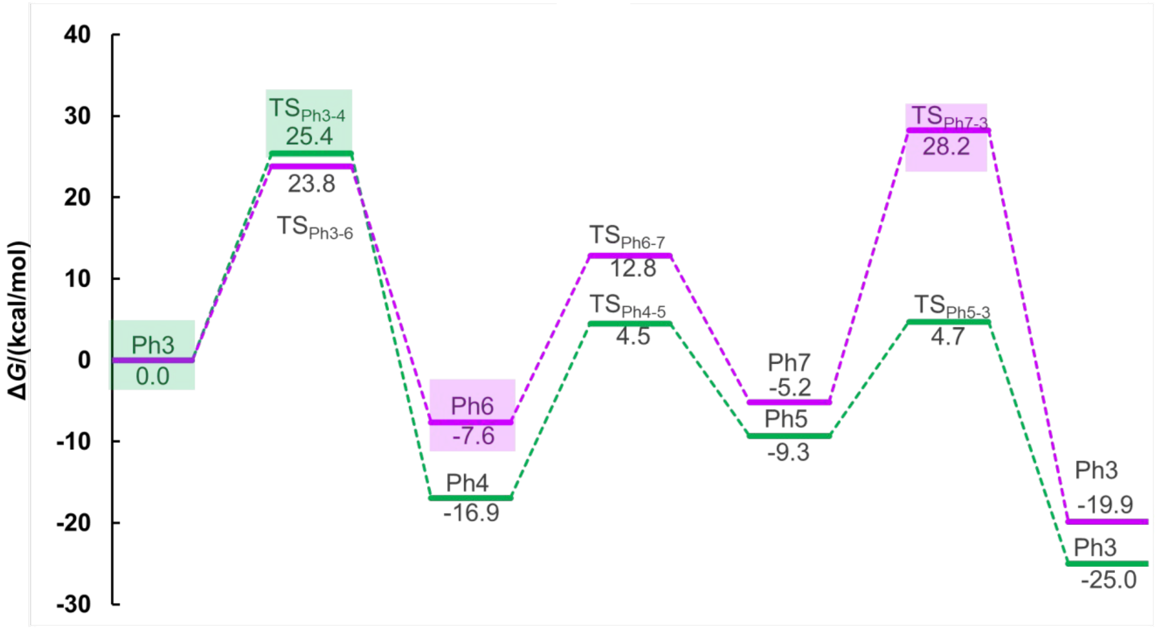

Figure S12. The free energy profile for the hydrogenation of 1,2-diphenylethyne to 1,2-diphenylethene (green line) and 1,2-diphenylethene to 1,2-diphenylethane (purple line) that catalyzed by **m3**. The TDI and TDTS are highlighted in the green or purple boxes along each pathway.

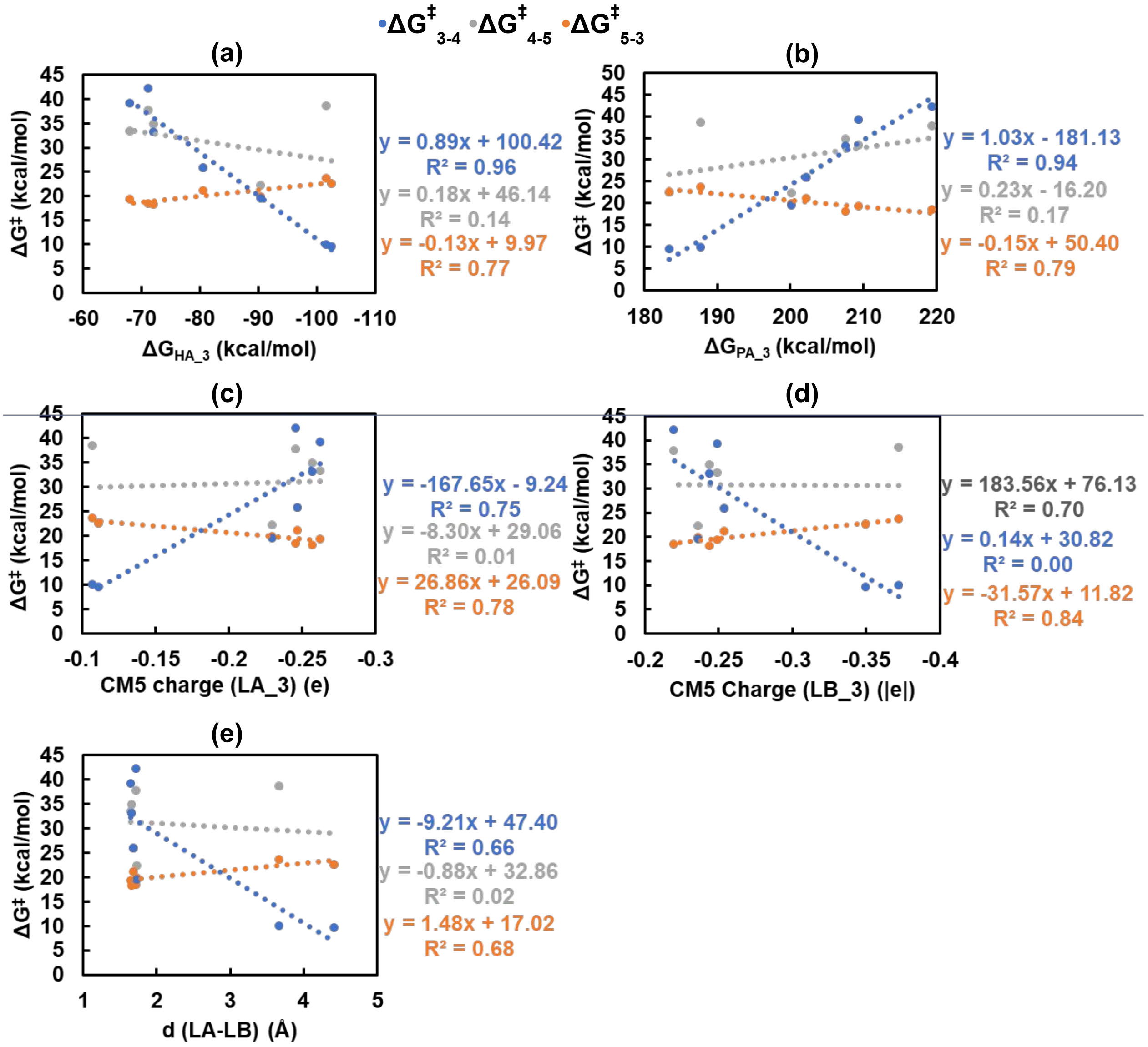

Figure S13. The free energies of activation for the hydrogenation of acetylene to ethene ($\Delta G^‡_{4\text{-}5}$, $\Delta G^‡_{5\text{-}3}$ and $\Delta G^‡_{3\text{-}4}$) catalyzed by FLP **a-g** as a function of (a) hydride attachment energy, (b) proton attachment energy, (c) CM5 charge of LA site, (d) CM5 charge of LB site, and (e) LA-LB linker distance.

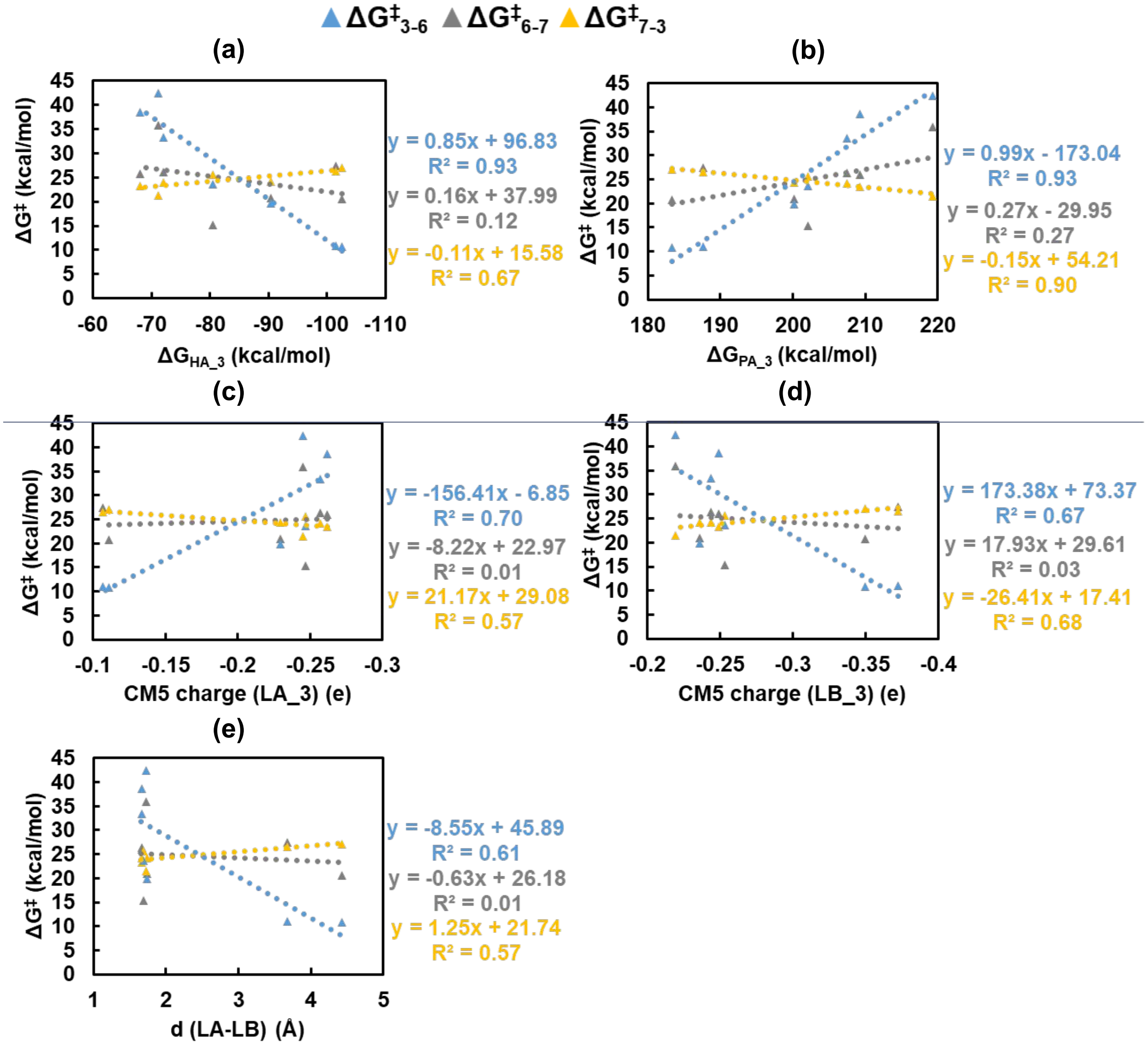

Figure S14. The free energies of activation the hydrogenation of ethene to ethane ($\Delta G^{\ddagger}_{4\text{-}5}$, $\Delta G^{\ddagger}_{5\text{-}3}$ and $\Delta G^{\ddagger}_{3\text{-}4}$) catalyzed by FLP **a-g** as a function of (a) hydride attachment energy, (b) proton attachment energy, (c) CM5 charge of LA site, (d) CM5 charge of LB site, and (e) LA-LB linker distance.

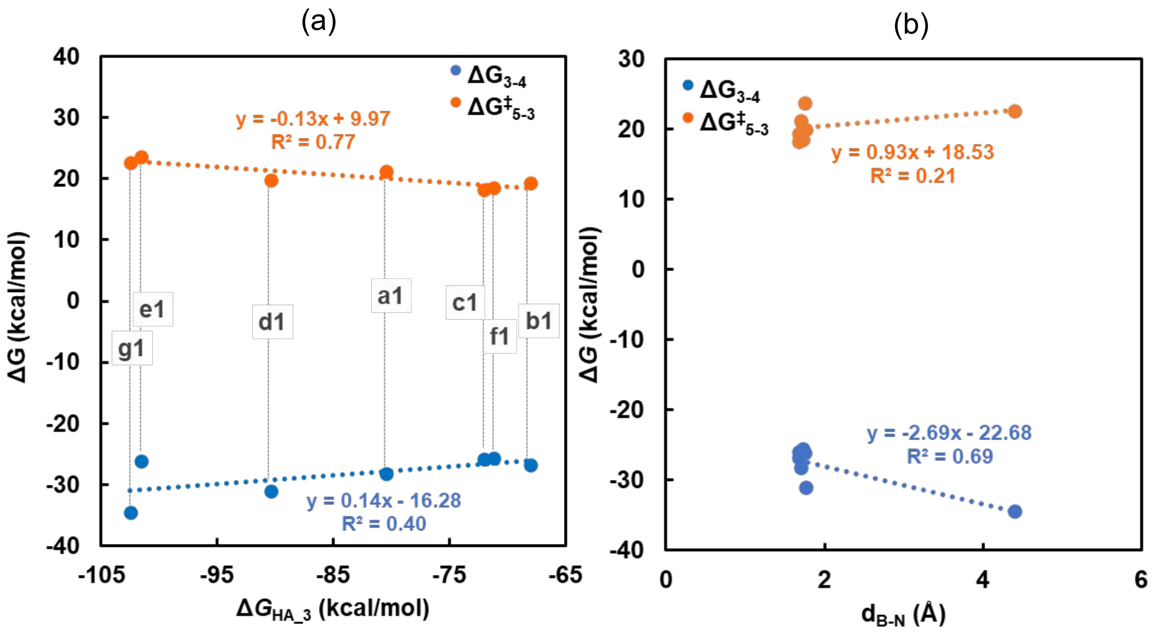

Figure S15. The correlation between the free energies of reaction for alkyne insertion (3→4, $\Delta G_{3\text{-}4}$) and the free activation energies for intramolecular protonation (5→3, $\Delta G^{‡}_{5\text{-}3}$) catalyzed by **a1-g1** and (a) hydride attachment energies ($\Delta G_{\mathrm{HA\_3}}$) of **a3-g3** and (b) the distance between LA and LB sites ($d_{\mathrm{B\text{-}N}}$).

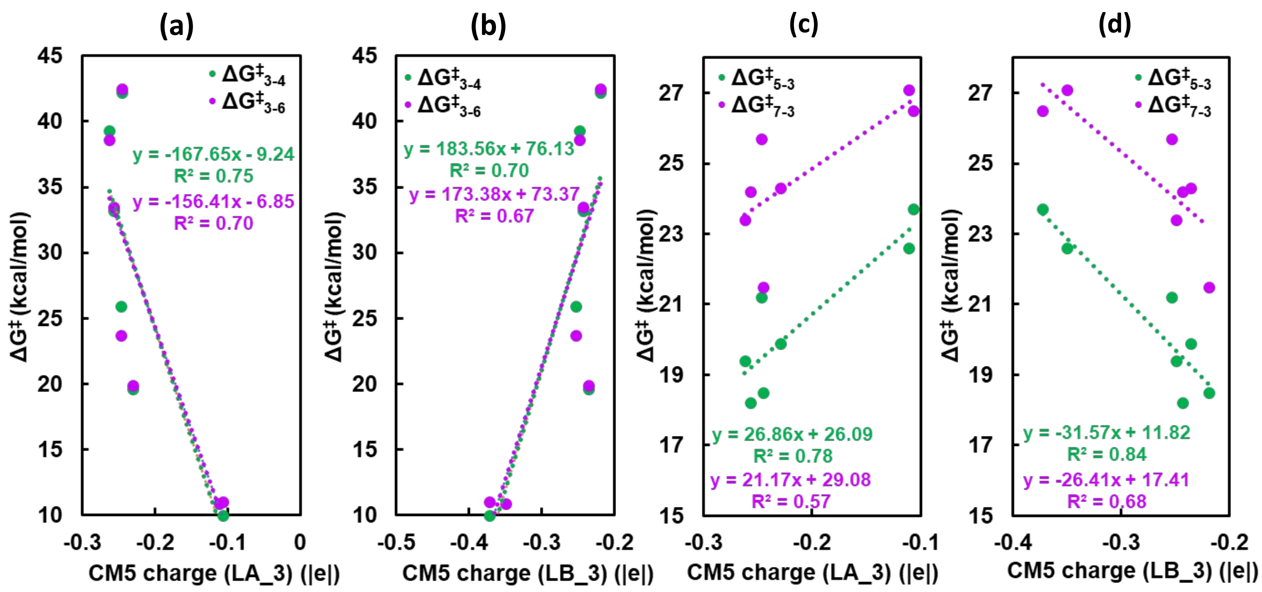

Figure S16. The activation free energies of acetylene/ethene insertion (**3→4/3→6**) and intramolecular protonation (**5→3/7→3**) as a function of CM5 charge of (a) Lewis acid site (LA_3), and (b) Lewis base site (LB_3).

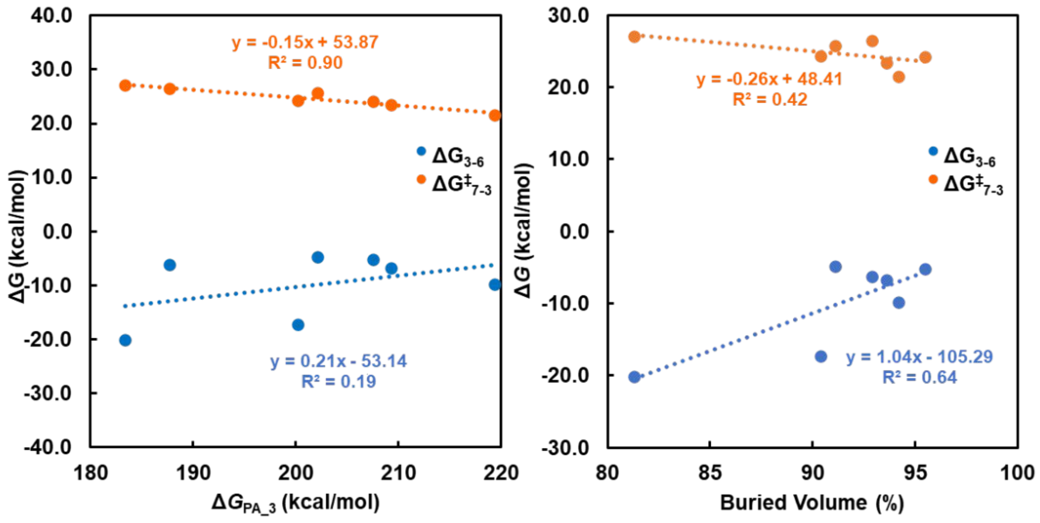

Figure S17. The correlation between the free energies of reaction for alkyne insertion (3→6, $\Delta G_{3\text{-}4}$) and the free activation energies for intramolecular protonation (7→3, $\Delta G^{\ddagger}_{5\text{-}3}$) catalyzed by **a1-g1** and (a) proton attachment energies ($\Delta G_{\mathrm{PA\_3}}$) of **a3-g3** and (b) the buried volume of FLP catalysts (**a1-g1**). (c) The correlation between $\Delta G_{\mathrm{PA\_3}}$ and CM5 charge of Lewis base site (N) of **a3-g3**.

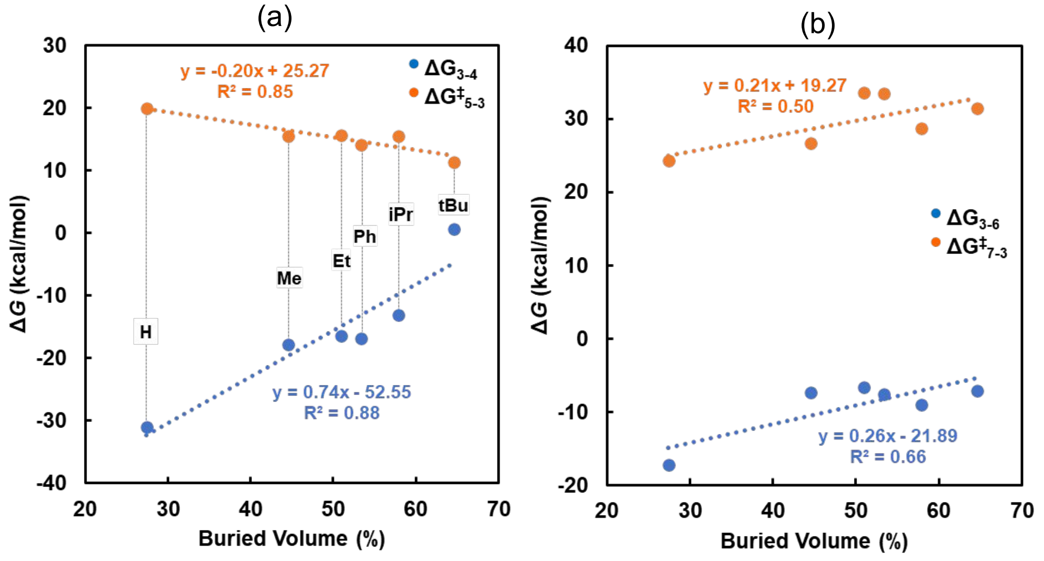

Figure S18. (a) The correlation between the free energies of reaction for alkyne insertion (3→4, $\Delta G_{3\text{-}4}$) and the free energies of activation for intramolecular protonation (5→3, $\Delta G^{\ddagger}_{5\text{-}3}$) catalyzed by **d1** and the buried volume of alkynes. (b) The correlation between the free energies of reaction for alkene insertion (3→6, $\Delta G_{3\text{-}6}$) and the free energies of activation for intramolecular protonation (7→3, $\Delta G^{\ddagger}_{7\text{-}3}$) catalyzed by **d1** and the buried volume of alkynes.

Table S1. The calculated TOF at 298.15 K and 1 atm using energetic span model for FLPs toward acetylene hydrogenation to ethene and ethene to ethane.

FLP	alkyne	$\Delta G^{\neq}_{C\equiv C}$ (kcal/mol)	$\Delta G^{\neq}_{C=C}$ (kcal/mol)	$TOF_{C\equiv C}$ (s^{-1})	$TOF_{C=C}$ (s^{-1})	$TOF_{C\equiv C}$/ $TOF_{C=C}$
a3	HC≡CH	31.9	28.7	2.6E-11	5.7E-9	4.5E-03
b3	HC≡CH	39.9	38.9	3.5E-17	1.9E-16	1.9E-01
c3	HC≡CH	40.7	40	9.1E-18	3.0E-17	3.1E-01
d3	HC≡CH	27.3	32.6	6.1E-08	7.9E-12	7.7E+03
e3	HC≡CH	42.9	41.7	2.2E-19	1.7E-18	1.3E-01
f3	HC≡CH	42.5	45.9	4.4E-19	1.4E-21	3.1E+02
g3	HC≡CH	30.4	35.8	3.2E-10	3.6E-14	9.1E+03
a3	CH_3−C≡C−CH_3	21	32.6	2.5E-03	7.9E-12	3.2E+08
a3	Et−C≡C−Et	21.2	36	1.8E-03	2.5E-14	7.1E10
a3	iPr−C≡C−iPr	22.3	29.1	2.8E-04	2.9E-09	9.7E+04
a3	tBu−C≡C−tBu	23.2	38.9	5.7E-5	1.9E-16	3.0E+11
a3	ph−C≡C−ph	21.6	35.8	9.1E-04	3.6E-14	2.6E+10

Table S2. The bond lengths of LA-LB (*d*(LA-LB)), the CM5 charges of LA and LB site, HOMO energies, LUMO energies and HOMO-LUMO gaps, hydride attachment energies (ΔG_{HA_1}) and (b) proton attachment energies (ΔG_{PA_1}) of **a1-g1**.

**FLPs**	***d*(LA-LB) (Å)**	**CM5 (LB)**	**CM5 (LA)**	**[a]ΔG_{HA_1} (kcal/mol)**	**[b]ΔG_{PA_1} (kcal/mol)**
**a1**	1.693	-0.246	-0.168	-96.68	204.04
**b1**	1.674	-0.238	-0.170	-86.54	211.54
**c1**	1.675	-0.233	-0.163	-90.56	206.69
**d1**	1.758	-0.230	-0.147	-106.90	199.19
**e1**	1.747	-0.234	-0.150	-97.13	200.69
**f1**	1.728	-0.215	-0.153	-92.09	210.94
**g1**	4.395	-0.350	-0.062	-112.70	185.52

a. $\Delta G_{HA\_1} = G(x1\_H^-) - G(x1) - G(H^-)$

b. $\Delta G_{PA\_1} = G(x1\_H^+) - G(x1) - G(H^+)$

Table S3. The bond lengths of LA-LB (*d*(LA-LB)), the CM5 charges of LA and LB site, HOMO energies, LUMO energies and HOMO-LUMO gaps, , hydride attachment energies (ΔG_{HA_3}) and (b) proton attachment energies (ΔG_{PA_3}) of **a3-g3**.

**FLPs**	***d*(LA-LB) (Å)**	**CM5 (LB)**	**CM5 (LA)**	**$^{a}\Delta G_{HA\_3}$ (kcal/mol)**	**$^{b}\Delta G_{PA\_3}$ (kcal/mol)**
**a3**	1.680	-0.254	-0.247	-80.50	202.13
**b3**	1.651	-0.249	-0.262	-68.01	209.28
**c3**	1.662	-0.244	-0.257	-72.01	207.50
**d3**	1.725	-0.236	-0.230	-90.45	200.17
**e3**	3.660	-0.372	-0.107	-101.59	187.66
**f3**	1.714	-0.219	-0.245	-71.17	219.34
**g3**	4.411	-0.350	-0.111	-102.54	183.37

a. $\Delta G_{HA\_3} = G(x3\_H^{-}) - G(x3) - G(H^{-})$

b. $\Delta G_{PA\_3} = G(x3\_H^{+}) - G(x3) - G(H^{+})$

Table S4. The Gibbs free energies of reaction and activation of each elementary step for the hydrogenation of alkyne to alkene for **a3-g3**. (x is a letter from a-g)

FLP	alkyne	ΔG_{x3-4}	ΔG_{x4-5}	ΔG_{x5-3}	$\Delta G^{‡}_{x3-4}$	$\Delta G^{‡}_{x4-5}$	$\Delta G^{‡}_{x5-3}$
a3	HC≡CH	-28.2	10.8	17.5	25.9	25.9	21.2
b3	HC≡CH	-26.8	20.5	6.3	39.3	33.4	19.4
c3	HC≡CH	-25.9	22.6	3.3	33.2	34.9	18.2
d3	HC≡CH	-31.0	7.5	23.6	19.6	22.3	19.9
e3	HC≡CH	-26.2	19.2	35.9	10.0	38.6	23.7
f3	HC≡CH	-25.6	24.0	1.6	42.2	37.8	18.5
g3	HC≡CH	-34.5	7.8	26.7	9.6	22.6	22.6
a3	CH_3−C≡C−CH_3	-17.8	5.5	12.3	19.5	19.1	15.5
a3	Et−C≡C−Et	-16.4	5.6	10.8	19.0	18.8	15.6
a3	iPr−C≡C−iPr	-13.1	6.9	6.2	19.8	17.7	15.5
a3	tBu−C≡C−tBu	1.8	4.9	-11.9	21.0	20.3	11.3
a3	ph−C≡C−ph	-16.9	7.6	9.3	27.7	21.4	14.1

Table S5. The Gibbs free energies of reaction and activation of each elementary step for the hydrogenation of alkene to alkane for **a3-g3**. (x is a letter from a-g)

FLP	alkyene	$\Delta G_{x3\text{-}6}$	$\Delta G_{x6\text{-}7}$	$\Delta G_{x7\text{-}3}$	$\Delta G^{\ddagger}_{x3\text{-}6}$	$\Delta G^{\ddagger}_{x6\text{-}7}$	$\Delta G^{\ddagger}_{x7\text{-}3}$
a3	$H_2C{=}CH_2$	-4.8	3.0	1.8	23.7	15.4	25.7
b3	$H_2C{=}CH_2$	-6.7	15.5	-8.8	38.6	26.0	23.4
c3	$H_2C{=}CH_2$	-5.2	15.9	-10.7	33.5	26.3	24.2
d3	$H_2C{=}CH_2$	-17.3	8.3	8.9	19.9	20.9	24.3
e3	$H_2C{=}CH_2$	-35.2	15.2	19.9	11.0	27.5	26.5
f3	$H_2C{=}CH_2$	-9.8	24.5	-14.6	42.5	36.0	21.5
g3	$H_2C{=}CH_2$	-20.1	8.7	11.4	10.9	20.7	27.1
a3	$CH_3{-}HC{=}CH{-}CH_3$	-7.4	6.0	1.4	21.1	19.5	26.6
a3	Et−HC=CH−Et	-6.7	2.3	4.3	19.3	20.6	33.6
a3	iPr−HC=CH−iPr	-9.0	0.4	8.6	13.6	20.1	28.7
a3	tBu−HC=CH−tBu	6.2	-5.6	-0.3	20.6	8.0	31.4
a3	ph−HC=CH−ph	-7.6	2.5	5.2	23.8	20.5	33.4

Table S6. Buried volume with H included.

**structures**	**Buried volume (%)**
**a1**	91.1
**b1**	93.6
**c1**	95.5
**d1**	90.4
**e1**	92.9
**f1**	94.2
**g1**	81.3
HC≡CH	27.4
$CH_3{-}C{\equiv}C{-}CH_3$	44.5
Et−C≡C−Et	51.0
iPr−C≡C−iPr	57.9
tBu−C≡C−tBu	64.6
ph−C≡C−ph	53.4
